# The hidden impact of alcohol on young victims: an analysis of alcohol-related police offences resulting in hospitalisation

**DOI:** 10.1186/s12889-024-17704-w

**Published:** 2024-01-17

**Authors:** Scott Anthony Sims, Gavin Pereira, Daniel Fatovich, David Preen, Melissa O’Donnell

**Affiliations:** 1 School of Population and Global Health, University of Western Australia, Perth, Australia; 2 Curtin School of Population Health, Curtin University, Perth, Australia; 3 enAble Institute, Curtin University, Perth, Australia; 4 Emergency Medicine, Royal Perth Hospital, University of Western Australia, Perth, Australia; 5 Centre for Clinical Research in Emergency Medicine, Harry Perkins Institute of Medical Research, Nedlands, Australia; 6 Australian Centre for Child Protection, University of South Australia, Adelaide, Australia

**Keywords:** Alcohol-related harm, Police incidents, Victims, Linked data, Under-ascertainment

## Abstract

**Background:**

Alcohol-related harm (ARH) is a significant public health concern affecting young individuals, particularly those involved in alcohol-related police incidents resulting in hospitalisation. However, the impact of alcohol on young victims remains under researched. This study aimed to identify the characteristics of offenders and victims involved in these incidents, analyse the types of offences, and understand the under-ascertainment of ARH in hospital records.

**Methods:**

A retrospective longitudinal study of 12–24-year-olds born between 1980 and 2005 was conducted using linked data from hospital admissions, emergency department presentations, and police incident records. Alcohol-related incidents were identified based on the attending officers’ opinions in the Western Australia Police’s Incident Management System (IMS). Logistic and log-binomial regression were utilised to analyse the factors associated with victimisation and under-ascertainment of ARH.

**Results:**

Our study included 22,747 individuals (11,433 victims and 11,314 offenders) involved in alcohol-related police incidents, with a small majority of victims being female (53%, *n* = 6,074) and a large majority of offenders being male (84.3%, *n* = 9,532). Most victims did not receive a diagnosis of ARH (71%, *n* = 760). Women were 10 times more likely to have been a victim in ARH police incidents and 2 times more likely to have an undiagnosed alcohol-related hospital admission than men. Victims and offenders predominantly came from disadvantaged areas and major cities. Aboriginal individuals were overrepresented as both offenders and victims. A significant proportion of individuals experienced emergency department presentations or hospital admissions, with head injuries being the most common. Assault causing bodily harm was the most prevalent offence resulting in hospitalisation (66%, *n* = 2,018).

**Conclusions:**

There is a noteworthy disparity between the quantity of hospital admissions attributed to alcohol-related incidents and the number of cases that are formally classified as ARH in the hospital system. This disparity highlights a more profound issue of substantial under-ascertainment or inadequate identification of ARH than previously acknowledged. Our findings justify the prioritisation of prevention strategies, beyond improvement in the documentation of alcohol-related hospitalisation. Considering the scale of the problem, and the underestimation of the burden of alcohol-related hospitalisation, a proportional increase in investment is necessary to achieve population-level reductions in ARH.

**Supplementary Information:**

The online version contains supplementary material available at 10.1186/s12889-024-17704-w.

## Background

Alcohol-related harm (ARH) continues to be a significant public health concern, particularly among young individuals who are disproportionately impacted by its detrimental effects [1–3]. The consequences of excessive alcohol consumption extend beyond the immediate physical and psychological effects experienced by the drinker [4]. Of particular concern is the impact that alcohol has on young victims, particularly those who become involved in alcohol-related police incidents resulting in hospitalisation. Alcohol use by perpetrators has been identified as a prevalent factor in physical and sexual assaults, with studies reporting that over half of these incidents involve alcohol [5]. Furthermore, alcohol has been implicated in child maltreatment incidents [6, 7] and the occurrence of road traffic crashes [8]. These findings emphasise the important need to understand the extent of ARH and its impact on young victims.

To comprehensively study ARH, researchers often rely on hospital administrative data, which offer valuable insights using International Classification of Disease (ICD) coding to record diagnostic information. However, a limitation arises regarding capturing the full scope of ARH. Hospital administrative data primarily focus on patients admitted with alcohol-related diagnoses, thus overlooking cases where the perpetrator, rather than the hospitalised victim, had been under the influence of alcohol [3, 9]. This limitation restricts the available diagnostic information, particularly within emergency department (ED) data collections, as ICD codes often provide only the proximate cause of the injury [10]. As a result, estimates of ARH are likely significant under representations of the true prevalence of alcohol-related hospitalisations and associated diagnoses [11, 12].

To address these limitations and enhance our understanding of ARH, alternative approaches that facilitate the examination of perpetrator characteristics warrant exploration [13]. Linking alcohol-related incident data collected by the Western Australia Police Force with hospital admission data presents an opportunity to gain deeper insights into the characteristics of both victims and offenders involved in alcohol-related police incidents. Such linkage enables the identification of hospital admissions lacking ARH diagnosis information when the offender was under the influence of alcohol.

This study aims to identify the characteristics of both offenders and victims involved in incidents with law enforcement related to alcohol consumption, and elucidate the specific nature of these incidents. Additionally, we seek to examine the attributes and injury conditions of individuals who were hospitalised subsequent to their participation in alcohol-related police incidents. Furthermore, we investigated the underreporting of ARH in hospital records to identify factors linked to instances where alcohol-related hospital admissions went unidentified. Together, this research will provide a comprehensive understanding of the types of offences that are more likely to lead to hospitalisation after a police-involved ARH incident. Focusing specifically on individuals aged 12–24 years, a critical developmental stage often associated with heavy episodic drinking [14, 15], this research seeks to inform the development of targeted interventions and shape policies designed to mitigate harm and prevent future alcohol-related injuries within this vulnerable population.

## Methods

### Design and participants

This was a retrospective longitudinal study of all children and young adults in Western Australia (WA) aged 12–24 years and born between 1980 and 2005 who were involved in an alcohol-related incident between July 2004 and June 2015. WA is a geographically large state, with approximately 75% of its population residing in the Perth metropolitan area. The state also includes vast and remote regions, which present unique challenges and opportunities for public health research, including issues related to access to healthcare, mental health, and environmental factors.

### Data sources

Population-level data for this study were obtained from the Emergency Department Data Collection (EDDC), Hospital Morbidity Data Collection (HMDC), Midwife Notification System (MNS), Department of Child Protection (DCP) data collection and merged with the Western Australia Police (WAPOL) offence data collection to form the cohort of this study. Using probabilistic linkage of common identifiers, such as name, address, and birth date, the data were linked by the Department of Health’s Data Linkage Branch in which extensive clerical review was also conducted as per their process, with a linkage quality of at least 97% [14, 15]. The identifiers were separated from the clinical or service information to maximise privacy during the linkage process, with only de-identified information provided to researchers.

The EDDC contains all presentations to EDs in WA and includes the patient’s principal diagnosis upon completion of the ED service event. The HMDC comprises all episodes of care for patients discharged from all public and private hospitals in WA, including up to 21 diagnostic codes and four external cause codes based on the International Classification of Diseases (ICD-10-AM). Administrative data from the MNS were used to provide birth characteristics for those who were born in WA, including maternal demographics such as age and ethnicity, as well as the child’s gestation period and birth weight. Child protection notifications were extracted from the Western Australian Child Protection Data collection for the period 1990–2015. WA Police provided incident data for both the victim and offender categorised by offence type and date of incident, where alcohol was identified (alcohol flag). A victim or offender involved in an incident was deemed to have a matching ED presentation if they visited the ED within one day of the offence date. An admission to the hospital was considered matching if it was within one day of either the matching ED presentation or offence date.

### Identification of alcohol-related harm

Hospital admissions were classified as alcohol-related if the following ICD-10-AM codes appeared in any of the diagnostic fields for a patient episode of care: F10, E24.4, G31.2, G62.1, G72.1 I42.1, K29.2, K70, K85.2, K86.0, R78.0, T50.6, T51, X45, X65, Y15, Y91, Z50.2, Z71.4, and Z72.1. A police incident may be flagged as alcohol-related in the WA Police Incident Management System (IMS) if the attending officer is of the opinion that alcohol was related in any way to the incident. These include but are not limited to the offender or victim’s level(s) of intoxication at the time of the incident and may include circumstances such as identifying open or used alcohol receptacles, smelling alcohol at the scene, observing an individual affected by alcohol or receiving alcohol-related information from victims, offenders or a third party, such as witnesses. The absence of any of these circumstances does not necessarily preclude that alcohol was a contributing factor to the incident. Flagging incidents as alcohol-related is a mandatory requirement in the incident report.

### Outcomes

Two model outcomes were analysed. The first focused on the risk of being a victim in an alcohol-related police incident compared to being the offender. The second model outcome examined individuals who were hospitalised following an alcohol-related incident but did not receive a coded diagnosis of ARH. This model was restricted to only ARH police incidents that resulted in a hospital admission to obtain a true indicator of under-ascertainment. We also identified specific offence types from alcohol-related incidents that were associated with hospitalisation. All hospitalisations refer to inpatient admissions and exclude inter-hospital transfers as these were regarded as part of the same episode of care.

### Demographic characteristics

Demographic characteristics such as age, sex, ethnicity, socio-economic status, and remoteness area were included in the regression modelling. The birth cohort year was included to account for the confounding caused by the skewed distribution of age. Demographic variables were obtained from the MNS data collection and police incident data. Ethnicity was derived by the WA Data Linkage Branch using a validated algorithm for any individual with at least one record in several WA government administrative datasets where Aboriginal status was recorded [16]. The term ‘Aboriginal’ includes all young people identifying as Aboriginal and/or Torres Strait Islander origin. The 2011 Socio-Economic Indexes for Areas (SEIFA) index, the Index of Relative Socio‐Economic Advantage and Disadvantage, is a statistical tool used in Australia to measure the relative socio-economic status of different geographic areas. This was used to compare variations in the proportion of identified alcohol‐related harm across socio‐economic levels in the two diagnosis methods. In our study, the index is represented as a ranking of areas in WA into quintiles, from least socio‐economically advantaged to most socio‐economically advantaged [17]. Remoteness area was defined using the Australian Statistical Geographic Standard Remoteness Area (ASGS‐RA), which is based on a measure of relative access to services and categorised into five classes of remoteness: major cities, inner regional, outer regional, remote, and very remote areas [18]. A hospital admission was classified as having been admitted through an emergency department if the ‘Source of Referral’ was an ED clinician or the mode of transport to hospital was by emergency ambulance.

### Statistical analysis

To quantify the proportion of hospitalised victims who had an alcohol-affected offender, descriptive statistics were performed for all demographic characteristics of the study cohort using both WA Police offence data and hospital admissions. Injury admissions related to single body regions based on any diagnostic code for injury, poisoning or other consequences of external cause ICD codes: S00-S99. A logistic regression model was employed to analyse the relationship between the victim/offender status and the hospital admission variable. Logistic regression is commonly used when the outcome variable is binary (e.g., hospital admission vs. no hospital admission) and is suitable for estimating odds ratios. However, when estimating the risk of offence types in individual models, a log-binomial model was used instead of a logistic regression model. This is because log-binomial models are more appropriate when the outcome is a rare event, as they directly estimate the relative risk. Variables selected for inclusion in the regression modelling was guided by considering known confounders, risk factors linked to ARH, and factors of clinical significance. Predictors of hospital admissions and potential confounders were adjusted for by inclusion in all models of the covariates sex, ethnicity, socio-economic status (SES), remoteness area, and birth cohort year. Overall model adequacy was assessed by global chi-square goodness of fit tests which showed the models as a whole fit significantly better than empty models. Model fit statistics showed lower values across all three criteria (AIC, BIC, -2 Log L, and QIC) for the models with covariates which indicated better representation of the data than the intercept only model. Individuals with multiple records were accounted for to ensure the analysis appropriately considered the correlation or dependence between observations from the same individual. Multicollinearity was assessed using correlation matrix for parameter estimators. The collinearity diagnostics indicated no significant multicollinearity issues among the predictor variables. A plot of predicted probabilities was performed to confirm the linearity assumption held for age at offence in the models. We addressed missing data by using complete case analysis because there was relatively low missingness among the relevant variables. Analyses were conducted with SAS V9.4 (SAS Institute, Cary, NC, USA).

### Ethics

#### Ethics approval

for this study was obtained from the WA Aboriginal Health Ethics Committee [Ref: 755], the University of WA Human Research Ethics Committee [Ref: RA/4/1/8822], and the Department of Health WA Human Research Ethics Committee [Ref: 2016/55].

## Results

A total of 22,747 young people (11,433 victims and 11,314 offenders) aged 12–24 were involved in alcohol-related police incidents from 2004 to 2015 (Table 1). Almost 1 in 10 victims (*n* = 1,070) were hospitalised following an alcohol-related police incident, with 71% of these admissions undiagnosed as ARH. Most victims were female (53.1%), while the majority of offenders were male (84.3%). Aboriginal young people were overrepresented as both victims (32.6%) and offenders (44.9%) compared to their proportion in the general population (4.5% of all 15–24 year olds in WA [19]). Victims and offenders were predominantly from the most disadvantaged areas. In addition, many incidents occurred in major cities, and there were no significant differences in remoteness area between victims and offenders. Most individuals involved in alcohol-related police incidents were between 17 and 24 years old. A significant proportion of individuals presented to EDs (35.6% of victims, 8.3% of offenders) or were admitted to the hospital (9.4% of victims, 1.7% of offenders), with a low proportion of these alcohol-related incidents being coded as alcohol-related in the hospital admission data (29.0% of victims, 55.3% of offenders). Of these hospitalisations, head injuries (72%) were the most common injury type, of which 35% were open wounds of the head and 25% were fractures of the skull and facial bones (Supplementary Table S2). A substantial proportion of individuals had prior involvement with the courts (42.7% of victims, 70.3% of offenders) and the child protection system (21.7% of victims, 26.8% of offenders), indicating potential underlying issues such as family violence, abuse, or neglect.

**Table 1:** Characteristics of offenders and victims of alcohol-related police incidents for 12–24-year old’s, 2004–2015

Characteristic	Victims		Offenders	
	N	%	N	%
**Total**	**11,433**	**100.0**	**11,314**	**100.0**
Gender				
Female	6,074	53.1	1,764	15.6
Male	4,829	42.2	9,532	84.3
Missing	530	4.6	18	0.2
Ethnicity				
Non-Aboriginal	7,703	67.4	6,239	55.1
Aboriginal	3,730	32.6	5,075	44.9
SEIFA				
1 Least advantaged	4,419	38.7	4,942	43.7
2	2,570	22.5	2,480	21.9
3	1,625	14.2	1,528	13.5
4	1,243	10.9	1,053	9.3
5 Most advantaged	1,022	8.9	748	6.6
Missing	554	4.9	563	5.0
Remoteness Area				
Major Cities	5,416	47.4	5,351	47.3
Inner Regional	853	7.5	855	7.6
Outer Regional	1,664	14.6	1,557	13.8
Remote	1,104	9.7	1,101	9.7
Very Remote	1,842	16.1	1,887	16.7
Missing	554	4.9	563	5.0
Age at police incident				
12–16	914	8.0	1,242	11.0
17–18	2,048	17.9	2,209	19.5
19–20	3,107	27.2	2,701	23.9
21–22	2,884	25.2	2,707	23.9
23–24	2,480	21.7	2,455	21.7
Prior illicit drug admission	516	4.5	719	6.4
Prior alcohol admission	1,182	10.3	1,691	15.0
Prior mental illness admission	540	4.7	467	4.1
Prior community mental health service	804	7.0	924	8.2
Prior courts involvement	4,878	42.7	7,957	70.3
Prior DCP notification	2,483	21.7	3,036	26.8
Prior DCP period of care	550	4.8	794	7.0
Presented to ED	4,074	35.6	944	8.3
**Admitted to hospital**	**1,070**	**9.4**	**188**	**1.7**
Diagnosed as ARH	310	29.0	104	55.3
Undiagnosed ARH	760	71.0	84	44.7

Note: SEIFA = Socio-Economic Index for Areas; ED = Emergency Department; DCP = Department of Child Protection

Characteristics associated with being a victim in alcohol-related police incidents are shown in Table 2. Model estimates indicate that several factors significantly contribute to an increased risk of being a victim in these incidents. Females were found to be 10.3 times more likely than males to be victims (OR: 10.34, 95% CI: 9.62–11.12), highlighting a gender disparity in alcohol-related victimisation. Furthermore, non-Aboriginal people had nearly double the risk of victimisation compared to Aboriginal people (OR: 1.96, 95% CI: 1.82–2.10). Medical factors were also influential, with individuals admitted to the hospital because of the incident having a higher likelihood of victimisation (OR: 1.99, 95% CI: 1.66–2.38). Additionally, those who presented to the ED were significantly more likely to be victims (OR: 7.50, 95% CI: 6.87–8.20), emphasising the high level of physical harm of victims involved in alcohol-related police incidents. The absence of prior alcohol-related hospital admissions was associated with a 1.57 times higher risk of victimisation (OR: 1.57, 95% CI: 1.43–1.72). However, the effect size was smaller for individuals with no prior notifications to the DCP (OR: 1.15, 95% CI: 1.07–1.25), indicating a comparatively weaker association. Individuals with no prior involvement in the courts had a 2.4 times higher risk of being a victim in alcohol-related police incidents (OR: 2.37, 95% CI: 2.21–2.53), suggesting that a lack of legal history is likely a factor contributing to the risk profile of individuals experiencing victimisation.

**Table 2:** Characteristics associated with victims of alcohol-related police incidents compared to being the offender, for individuals aged 12–24 between 2004–2015

Characteristic	Unadjusted OR (95% CI)	Adjusted OR (95% CI)^a^
Sex		
Female	7.11 (6.70, 7.56)***	10.34 (9.62, 11.12)***
Male	REF	REF
Ethnicity		
Non-Aboriginal	1.63 (1.55, 1.72)***	1.96 (1.82, 2.10)***
Aboriginal	REF	REF
Admitted to hospital		
No	REF	REF
Yes	6.28 (5.40, 7.35)***	1.99 (1.66, 2.38)***
Presented to ED		
No	REF	REF
Yes	5.94 (5.53, 6.38)***	7.50 (6.87, 8.20)***
Prior ARH admission		
No	1.51 (1.40, 1.62)***	1.57 (1.43, 1.72)***
Yes	REF	REF
Prior DCP notification		
No	1.25 (1.18, 1.32)***	1.15 (1.07, 1.25)**
Yes	REF	REF
Prior courts involvement		
No	3.20 (3.04, 3.37)***	2.37 (2.21, 2.53)***
Yes	REF	REF

^a^ Multivariate model adjusted for all listed characteristics, including age at police incident and birth cohort year to account for differing length of follow-up time

* *p* < 0.05; ** *p* < 0.01; *** *p* < 0.0001

The focus of Table 3 is on alcohol-related police incidents resulting in hospital admissions that were not diagnosed as being alcohol related. The absence of an ARH diagnosis can occur if the admitted victim is not alcohol affected (but the offender was), or alcohol was not related to the presenting complaint. The risk estimates therefore demonstrate the association between these incidents and the under reporting of ARH hospital admissions. Specifically, victims of alcohol-related police offences were 2.6 times more likely than offenders to have hospital admissions without an alcohol-related diagnosis (OR: 2.57, 95% CI: 1.86–3.58). Gender disparities were also observed, with females involved in alcohol-related police incidents being 1.4 times more likely than males to undergo hospital admissions without an alcohol-related diagnosis (OR: 1.42, 95% CI: 1.07–1.90). Among the other characteristics examined, ethnicity wasn’t significantly linked to undiagnosed alcohol-related hospital admissions. Similarly, a patient’s history of ED presentations, alcohol-related hospital admissions, notifications to the DCP, or involvement with courts, had no significant associations with undiagnosed alcohol-related hospital admissions. However, having no prior alcohol-related hospital admission was strongly correlated with the absence of an ARH diagnosis in the admission resulting from the alcohol-related police incident. These results show that there are characteristics associated with young people that are more likely to not be captured in hospital administrative data as being alcohol-related, and therefore contribute to the under-ascertainment of ARH.

**Table 3:** Risk of undiagnosed alcohol-related hospital admission resulting from alcohol-related police incidents for individuals aged 12–24 admitted between 2004–2015

Characteristic	Unadjusted OR (95% CI)	Adjusted OR (95% CI)^a^
Sex		
Female	1.39 (1.09, 1.78)**	1.42 (1.07, 1.90)*
Male	REF	REF
Ethnicity		
Non-Aboriginal	1.15 (0.91, 1.45)	1.29 (0.96, 1.73)
Aboriginal	REF	REF
Presented to ED		
No	1.83 (0.79, 5.02)	2.28 (0.93, 6.47)
Yes	REF	REF
Prior ARH admission		
No	2.03 (1.52, 2.70)***	1.69 (1.23, 2.32)**
Yes	REF	REF
Prior DCP notification		
No	1.01 (0.77, 1.31)	0.90 (0.66, 1.24)
Yes	REF	REF
Prior courts involvement		
No	1.54 (1.22, 1.95)**	1.22 (0.94, 1.57)
Yes	REF	REF
Incident involvement		
Victim	2.95 (2.16, 4.03)***	2.57 (1.86, 3.58)***
Offender	REF	REF

^a^ Multivariate model adjusted for all listed characteristics, including age at police incident and birth cohort year to account for differing length of follow-up time

* *p* < 0.05; ** *p* < 0.01; *** *p* < 0.0001

Among victims aged 12–24 years between 2004 and 2015, Table 4 provides an overview of the top alcohol-related police offence types that led to both ED presentations and hospital admissions. Assault causing bodily harm was the most prevalent offence type that resulted in a hospitalisation, with 2,018 (65.6%) presentations and 473 (15.4%) admissions from a total of 3,074 victims. Common assault ranked second, with 1,063 (23.0%) ED presentations and 157 (3.4%) hospital admissions; however, this offence type impacted the most victims (*n* = 4,614). Wounding and grievous bodily harm were offence types where most ED presentations also resulted in a hospital admission. Offences such as dangerous driving causing grievous bodily harm, robbery, and deprivation of liberty had high ED presentation rates but lower hospital admission rates. Sexual offences, including sexual penetration without consent, demonstrated notable ED presentation rates (38.0%) compared to hospital admissions (5.6%).

**Table 4:** Prevalence of hospitalisations among victims of the top 20 alcohol-related police offence types for individuals aged 12–24 years, 2004–2015

Police offence types		Victims of offence type	Victim admitted to hospital		Victim presented to ED	
		N	n	%	n	%
1	*Assault causing bodily harm*	3,074	473	15.4	2,018	65.6
2	*Common assault*	4,614	157	3.4	1,063	23.0
3	*Wounding*	790	196	24.8	687	87.0
4	*Grievous bodily harm*	233	175	75.1	213	91.4
5	*Dangerous driving causing grievous bodily harm*	116	83	71.6	107	92.2
6	*Robbery*	294	15	5.1	77	26.2
7	*Damage*	718	12	1.7	65	9.1
8	*Deprivation of liberty*	131	17	13.0	60	45.8
9	*Criminal damage*	555	3	0.5	59	10.6
10	*Breach violence restraining order*	399	14	3.5	54	13.5
11	*Stealing*	529	8	1.5	53	10.0
12	*Threats to harm, take control of conveyance or building*	315	10	3.2	50	15.9
13	*Robbery in circumstances of aggravation*	129	8	6.2	43	33.3
14	*Burglary and commit*	226	8	3.5	41	18.1
15	*Sexual penetration without consent*	108	6	5.6	41	38.0
16	*Assault serious*	119	7	5.9	39	32.8
17	*Breach police restraining order*	364	10	2.7	39	10.7
18	*Act intended to cause grievous bodily harm or prevent arrest*	28	17	60.7	23	82.1
19	*Going armed in public to cause fear*	134	3	2.2	20	14.9
20	*Act or omission with intent to harm and causing bodily harm*	16	6	37.5	14	87.5
***TOTAL***		**11,433**	**1,070**	**9.4**	**4,074**	**35.6**

Some offence types vary in proportions between age groups of victims (Supplementary Table S3). For example, in the 12–16 age group, the most common offence types resulting in ED presentations and hospital admissions were assault causing bodily harm (59.8%), common assault (17.6%), and wounding (86.8%). However, in the 19–20 age group, the most common offence types were assault causing bodily harm (68.7%), common assault (24.8%), and wounding (84.6%). While the same offence types appear in both age groups, their percentages differ significantly. Similarly, in the 17–18 age group, the most common offence types were assault causing bodily harm (68.1%), common assault (24.8%), and wounding (88.0%), while in the 21–22 age group, the most common offence types were assault causing bodily harm (63.7%), common assault (21.9%), and wounding (87.7%). Again, there are some differences in the percentages of these offence types between these two age groups. Overall, the offence types that vary the most between age groups are those related to physical violence, such as assault causing bodily harm, wounding, and grievous bodily harm. The proportions of these offence types tend to increase in older age groups, indicating that alcohol-related violence becomes more severe as young people age.

**Fig. 1:**
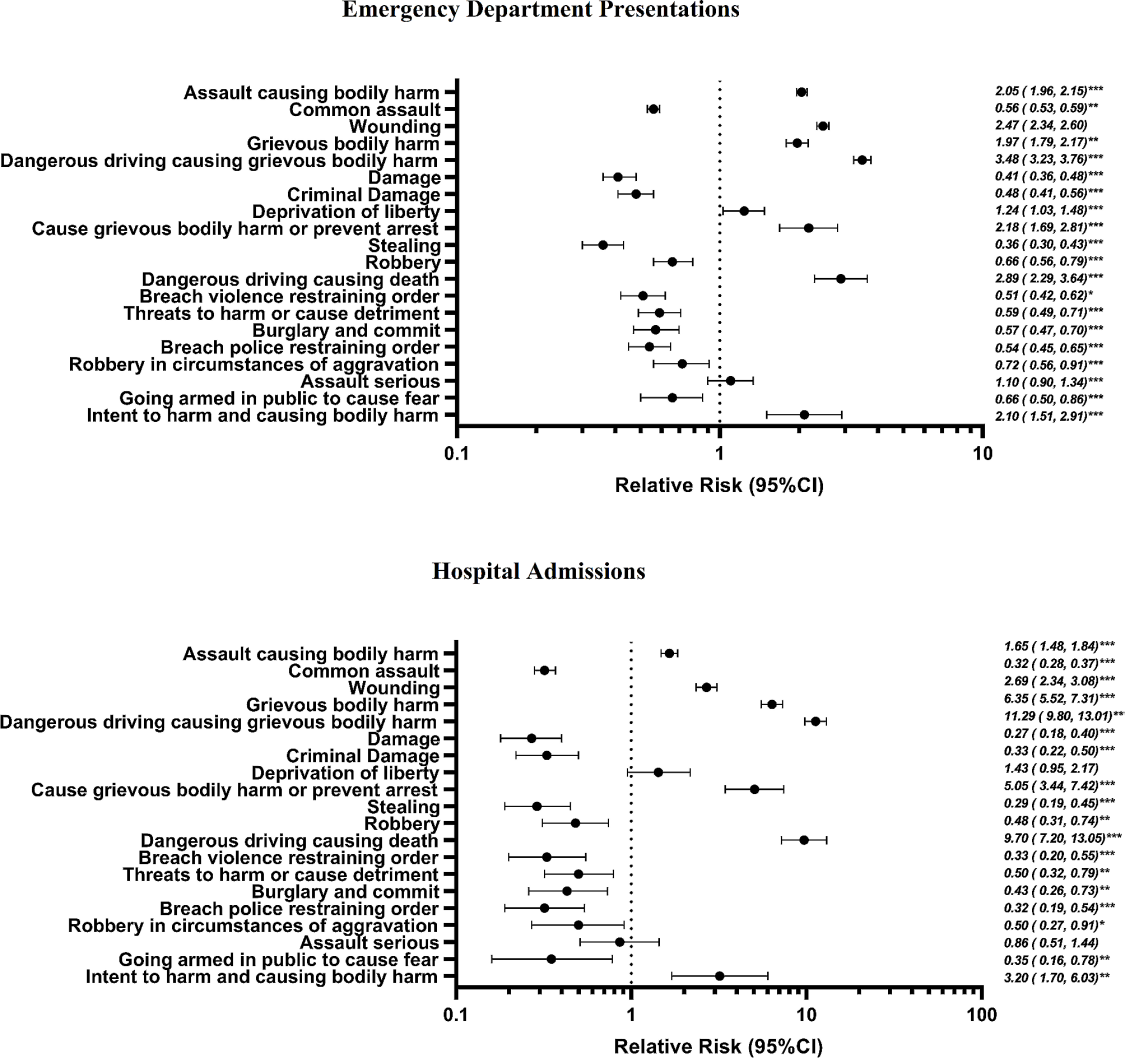
Q3Offence types most at risk of resulting in an ED presentation or hospital admission, young people aged 12–24 years, 2004-2015a,b. (a Adjusted for gender, ethnicity, SEIFA, remoteness area, and birth cohort. b * p < 0.05; ** p < 0.01; *** p < 0.0001). (Note: each relative risk represents an individual model containing a single offence type exposure, reference group = not having that offence type)

Figure 1 illustrates the risk of an ED presentation or hospital admission for different offence types, with the reference group being individuals not involved in that specific offence type. Each risk estimate was adjusted for confounding factors. This analysis provides valuable insights into the varying risks associated with different offence types in terms of ED presentations and hospital admissions, particularly emphasizing the severity of outcomes for young victims involved in alcohol-related incidents. In comparison to all other offence types combined, offences related to dangerous driving causing grievous bodily harm are 3.5 times more likely to result in an ED presentation. Similarly, dangerous driving causing death offences are 2.9 times more likely to lead to an ED presentation, while wounding offences are 2.5 times more likely. Regarding hospital admissions, offences related to dangerous driving causing grievous bodily harm have an 11.3 times higher likelihood of resulting in a hospital admission compared to all other offence types combined. Dangerous driving causing death offences is 9.7 times more likely to lead to a hospital admission, and grievous bodily harm offences are 6.3 times more likely.

## Discussion

Our study examined the under-identification of ARH among young people in Western Australia with a novel focus on victims involved in alcohol-related police incidents and the risks associated with different offence types. This emphasis on the hidden impact of alcohol on youth, especially victims, is important as most ARH research typically centres on the drinker themselves rather than the victims of their actions. Previous research has found a correlation between alcohol-related presentations in the emergency department and incidents attended by the police in a specific region of Australia [20]. Another Australian study further supports this relationship, demonstrating a short-term temporal association between emergency department attendances for alcohol problems and assaults reported to the police in New South Wales, Australia [21]. However, our study further elucidates the relationship between police incidents and hospitalisations by identifying the admissions not recognised as ARH despite resulting from an alcohol related incident. Internationally, the closest study addressing this underestimation was conducted in the United States, examining the underreporting of driver alcohol involvement in police and hospital records [22]. The authors analysed the proportion of US non-fatal crashes that involved alcohol and assessed how well police and hospitals detected this involvement. Similar to our study, the authors concluded that there is a need for improved communication between police and hospitals regarding alcohol involvement.

Our findings significantly contribute to the conversation around ARH by providing critical insights into the characteristics of both offenders and victims involved in alcohol-related police incidents. We found that approximately 71% of victims who were hospitalised due to an alcohol-related incident did not receive an ICD coding diagnosis of ARH. In particular, young victims, women, and those without prior ARH admissions were more likely to go unrecognised for ARH. This is most likely because it was the offender who was alcohol affected, and not the victim, and hence the hospital only diagnoses the presenting complaints of the individual admitted. Our study demonstrates the importance of linking police and hospital admission data, as it revealed that the accurate identification of ARH in female hospitalisation records would be missed without police data. This indicates that extending data linkage to other sources may also result in missed identification of ARH cases among women.

While our study highlights the overrepresentation of Aboriginal peoples as both victims and offenders, the issue of ARH becomes even more important when viewed within a global context. Indigenous populations worldwide consistently face higher rates of ARH [23, 24], often linked to the legacy of colonisation and the subsequent intergenerational transmission of problem behaviours [25, 26]. This emphasises the urgent need for culturally appropriate interventions to address the disproportionately high rates of ARH among Aboriginal adolescents. Additionally, our research also revealed that the majority of victims and offenders resided in major inner-city areas and came from disadvantaged backgrounds. This pattern aligns with international studies demonstrating that lower socio-economic groups bear a disproportionate burden from alcohol-related harms [27], underscoring the necessity for interventions tailored to socio-economic factors in addressing and mitigating ARH.

Our research revealed that over half of the victims were females, and a significant proportion of the offenders were males aged 18–24 years, consistent with previous studies demonstrating young males, especially those who drink heavily, are associated with increased likelihood of violence [28, 29]. The disparity between the males who largely constitute the offenders and females who form most of the victims found in our study also aligns with existing international literature. According to a report by the World Health Organisation, men are more likely to drink alcohol and subsequently engage in harmful behaviour, leading to the victimisation of others, predominantly women [30]. The prevalence of female victims and the vulnerability they face underline the need for targeted interventions to address gender disparities in alcohol-related victimisation.

The substantial number of victims with a history of court involvement and child protection notifications points to underlying issues such as family violence, abuse, or neglect, intensifying the impact on these individuals. Offences leading to hospitalisation, such as assault causing bodily harm and intentional self-harm, highlight the diverse risks associated with emergency department presentations and hospital admissions, providing crucial insights into the severity of outcomes for young victims. This understanding is vital for formulating targeted interventions and policies aimed at reducing harm and preventing alcohol-related injuries among this population.

Existing research has consistently shown associations between alcohol use by perpetrators and various forms of harm, including physical and sexual assaults, child maltreatment, and road traffic crashes [27, 28]. While ARH researchers typically rely on hospital administrative data with ICD coding for comprehensive diagnostic information, our study suggests that most estimates of ARH likely underrepresent the true proportion of alcohol-related hospitalisations and associated diagnostic information. The significance of our study lies in its specific focus on young victims hospitalised due to alcohol-related offences, a subject that has not received extensive investigation in previous research. These findings underscore the importance of healthcare providers remaining vigilant about the potential impact of alcohol on young victims presenting with injuries resulting from alcohol-related police incidents.

The findings of this study have important implications for policy and practice. First, the study highlights the need for targeted interventions to reduce ARH among young people, particularly males aged 18–24 years, as previously evidenced [31]. Our results showed likely historical issues of prior ARH, child protection, and court involvement for this cohort of offenders. Second, the study sheds light on the under reporting of ARH by linking it with alcohol-related police incident records and the need for more comprehensive diagnostic information to be recorded when ARH is suspected. Whilst it is not the expectation or responsibility of under resourced EDs to collect information on offenders, future research needs to be aware of this under-ascertainment for a more accurate estimate of the proportion of hospital admissions related to alcohol use [32]. This could entail innovative data analysis techniques such as machine learning and natural language processing to enhance ED data quality. For instance, the application of machine learning may help address the underdiagnosis of ARH and support those in recovery for ARH [33], as well as helping clinical staff identify and treat ARH patients [34]. Finally, the study’s findings underline the necessity for greater collaboration between health services and law enforcement agencies in identifying and supporting victims of ARH. Future research areas should focus on improving the ascertainment of ARH, developing targeted interventions for high-risk groups, strengthening the collaboration of health services and justice agencies to examine holistic approaches to the management and prevention of alcohol related harm. It is also necessary to utilise innovative data collection methods to enable us to monitor the effectiveness of alcohol related prevention, interventions, and policy responses.

### Limitations

Several limitations should be acknowledged. The study focused solely on hospital admissions resulting from police offences, potentially missing other alcohol-related hospital admissions. Since the police offence data only included incidents related to alcohol, there is a possibility that unidentified drugs, in addition to alcohol, may have played a role in the resulting hospitalisations. Additionally, the study’s geographical scope was limited to Western Australia, limiting the generalisability of the findings to other contexts. Last, the study specifically targeted young people aged 12–24 years, and therefore, the findings may not be applicable to other age groups.

## Conclusions

This study has provided important insights into the hidden impact of alcohol on young victims involved in alcohol-related police incidents. The findings suggest the need for targeted interventions and improved integration of police and other health care data and further exploration of alcohol’s impact on different age groups and settings to develop a more comprehensive understanding of this issue. Future research utilising hospital administrative data must consider the under-ascertainment of ARH, especially concerning victims of ARH, which cannot currently be identified unless linked data are used.

### Electronic supplementary material

Below is the link to the electronic supplementary material.

Supplementary Material 1

Supplementary Material 2

## Data Availability

The data that support the findings of this study are available from the WA Department of Health’s Data Linkage Branch, but restrictions apply to the availability of these data, which were used under license for the current study, and so are not publicly available. Data are however available from the authors upon reasonable request and with permission of Department of Health WA Human Research Ethics Committee and the Western Australian Aboriginal Human Research Ethics Committee. Note: The corresponding author has a signed ‘declaration of confidentiality’ agreement between the WA Department of Health that prevents unauthorised use, access, modification, or disclosure of the data by individuals not within the approved research project.
