# Supplementary material for: The hidden impact of alcohol on young victims: an analysis of alcohol-related police offences resulting in hospitalisation
Source: BMC Public Health. 2024 Jan 17;24:206. doi: 10.1186/s12889-024-17704-w (PMC10792924; doi:10.1186/s12889-024-17704-w)
Supplement: Supplementary file 1 — Supplementary Material 1 [file 12889_2024_17704_MOESM1_ESM.pdf]

Supplementary material for *The hidden impact of alcohol on young victims: an analysis of alcohol-related police offences resulting in hospitalisation.*

Table S1. Alcohol-related harm ICD-10-AM code categories and descriptions

ICD-10-AM	Description of alcohol-related hospitalisations
**E24.4**	Alcohol-induced pseudo-Cushing's syndrome
**E52**	Alcoholic pellagra *[if assigned with a code F10.x]*
**F10**	Mental and behavioural disorders due to use of alcohol
**G31.2**	Degeneration of nervous system due to alcohol
**G40.5**	Epileptic seizures related to alcohol
**G62.1**	Alcoholic polyneuropathy
**G72.1**	Alcoholic myopathy
**I42.6**	Alcoholic cardiomyopathy
**K29.2**	Alcoholic gastritis
**K70**	Alcoholic liver disease
**K85**	Alcohol pancreatitis *[if assigned with a code F10.x] [1999-Jul-01 to 2006-Jun-30]*
**K85.2**	Alcohol-induced acute pancreatitis *[2006-Jul-01 onwards]*
**K86.0**	Alcohol-induced chronic pancreatitis
**O35.4**	Maternal care for (suspected) damage to fetus from alcohol
**R78.0**	Finding of alcohol in blood
**T51.0**	Toxic effect: Ethanol
**T51.1**	Toxic effect: Methanol
**T51.9**	Toxic effect: Alcohol, unspecified
**X45**	Accidental poisoning by and exposure to alcohol
**X65**	Intentional self-poisoning by and exposure to alcohol
**Y15**	Poisoning by and exposure to alcohol, undetermined intent
**Y90**	Evidence of alcohol involvement determined by blood alcohol level *[excl. Y90.9]*
**Z71.4**	Alcohol abuse counselling and surveillance
**Z72.1**	Problems related to lifestyle - Alcohol use

Note: Codes selected as being appropriate for harm due to consumption of alcohol were based on clinical coding advice received from the Western Australian Clinical Coding Authority (WACCA). Z codes related to historic conditions were excluded. ICD-10-AM:International Statistical Classification of Diseases and Related Health Problems, Tenth Revision, Australian Modification

Table S2 ICD-10-AM injury groups assigned to hospital admissions following an alcohol-related police incidents for 12–24-year olds, 2004-2015.

Injury resulting from alcohol-related police incident	***Hospital admissions***		***Admitted through ED***
ICD-10-AM coding classification	n	%	%
Injuries to the head (S00-S09)	959	71.2	92.7
Superficial injury head (S00)	302	22.4	95.4
Open wound of head (S01)	472	35.1	94.3
Fracture of skull and facial bones (S02)	341	25.3	89.4
Dislocation, sprain and sprain of joints and ligaments of head (S03)	27	2.0	96.3
Injury of cranial nerves (S04)	12	0.9	83.3
Injury of eye and orbit (S05)	65	4.8	92.3
Intracranial injury (S06)	276	20.5	97.1
Crushing injury of head (S07)	0	0.0	-
Traumatic amputation of part of head (S08)	7	0.5	71.4
Other unspecified injuries of head (S09)	148	11.0	96.6
Injuries to the neck (S10-S19)	121	9.0	95.0
Injuries to the thorax (S20-S29)	172	12.8	96.5
Injuries to the abdomen, lower back, lumbar spine, and pelvis (S30-S39)	161	12.0	92.5
Injuries to the shoulder and upper arm (S40-S49)	148	11.0	93.9
Injuries to the elbow and forearm (S50-S59)	170	12.6	92.9
Injuries to the wrist and hand (S60-S69)	184	13.7	90.2
Injuries to the hip and thigh (S70-S79)	77	5.7	93.5
Injuries to the knee and lower leg (S80-S89)	134	10.0	96.3
Injuries to the ankle and foot (S90-S99)	47	3.5	89.4
Total injuries related to single body regions (S00–S99)	1,243	92.3	92.4
Total admissions	1,346	100.0	91.4

Table S3 Leading alcohol-related police offence types that result in hospitalisation of victims aged 12-24 years, 2004-2015.

	Offence type by age group	ED presentation		Hospital admission		Total victims
		n	%	n	%	N
	**Victim age 12-16**	**257**	**28.1**	**49**	**5.4**	**914**
1	Assault causing bodily harm	113	59.8	25	13.2	189
2	Common assault	72	17.6	10	2.4	410
3	Wounding	33	86.8	5	13.2	38
4	Sexual penetration of child aged 13-15	11	13.8	1	1.3	80
5	Dangerous driving causing grievous bodily harm	10	90.9	6	54.5	11
6	Aggravated sexual penetration without consent	7	41.2	1	5.9	17
7	Robbery	7	13.0	0	0.0	54
8	Robbery in circumstances of aggravation	6	26.1	0	0.0	23
9	Sexual penetration without consent	6	33.3	0	0.0	18
10	Stealing	6	10.7	0	0.0	56
	**Victim age 17-18**	**797**	**38.9**	**221**	**10.8**	**2,048**
1	Assault causing bodily harm	374	68.1	80	14.6	549
2	Common assault	213	24.8	35	4.1	860
3	Wounding	125	88.0	33	23.2	142
4	Grievous bodily harm	52	91.2	43	75.4	57
5	Dangerous driving causing grievous bodily harm	35	89.7	31	79.5	39
6	Robbery	27	30.0	6	6.7	90
7	Robbery in circumstances of aggravation	18	36.7	3	6.1	49
8	Stealing	13	15.9	1	1.2	82
9	Damage	11	9.8	2	1.8	112
10	Criminal damage	9	13.6	1	1.5	66
	**Victim age 19-20**	**1,156**	**37.2**	**285**	**9.2**	**3,107**
1	Assault causing bodily harm	574	68.7	125	15.0	836
2	Common assault	329	24.8	38	2.9	1,325
3	Wounding	193	84.6	56	24.6	228
4	Grievous bodily harm	64	90.1	54	76.1	71
5	Dangerous driving causing grievous bodily harm	28	93.3	20	66.7	30
6	Deprivation of liberty	24	57.1	4	9.5	42
7	Robbery	19	28.8	5	7.6	66
8	Stealing	18	13.3	4	3.0	135
9	Breach violence restraining order	17	15.9	2	1.9	107
10	Damage	16	8.2	4	2.0	196
	**Victim age 21-22**	**1,008**	**35.0**	**289**	**10.0**	**2,884**
1	Assault causing bodily harm	520	63.7	132	16.2	816
2	Common assault	240	21.9	44	4.0	1,096
3	Wounding	186	87.7	58	27.4	212
4	Grievous bodily harm	50	92.6	43	79.6	54
5	Dangerous driving causing grievous bodily harm	21	95.5	19	86.4	22
6	Criminal damage	20	10.9	0	0.0	184
7	Threats to harm, take control of building, or cause detriment	17	20.0	4	4.7	85
8	Breach violence restraining order	15	11.1	5	3.7	135
9	Deprivation of liberty	14	42.4	3	9.1	33
10	Burglary and commit	13	23.2	5	8.9	56
	**Victim age 23-24**	**856**	**34.5**	**226**	**9.1**	**2,480**
1	Assault causing bodily harm	437	63.9	111	16.2	684
2	Common assault	209	22.6	30	3.3	923
3	Wounding	150	88.2	44	25.9	170
4	Grievous bodily harm	43	95.6	33	73.3	45
5	Damage	24	11.8	4	2.0	204
6	Breach violence restraining order	17	16.8	7	6.9	101
7	Burglary and commit	16	22.9	2	2.9	70
8	Breach police restraining order	15	15.3	3	3.1	98
9	Criminal damage	15	10.4	2	1.4	144
10	Assault serious	13	21.7	0	0.0	60
	TOTAL victims aged 12-24	***4,074***	***35.6***	***1,070***	***9.4***	***11,433***
