# Supplementary material for: The hidden impact of alcohol on young victims: an analysis of alcohol-related police offences resulting in hospitalisation
Source: BMC Public Health. 2024 Jan 17;24:206. doi: 10.1186/s12889-024-17704-w (PMC10792924; doi:10.1186/s12889-024-17704-w)
Supplement: Supplementary file 2 — Supplementary Material 2 [file 12889_2024_17704_MOESM2_ESM.pdf]

Supplementary material: Model fit and assumption results from SAS V9.4

TABLE 2

Model fit statistics for model in Table 2:

Model Fit Statistics		
**Criterion**	**Intercept Only**	**Intercept and Covariates**
AIC	34834.141	24862.449
SC	34842.273	24943.766
-2 Log L	34832.141	24842.449

This demonstrates that the model including covariates is considered superior in terms of goodness of fit and model complexity. Lower values across all three criteria (AIC, BIC, and -2 Log L) for the model with covariates indicate that it provides a better representation of the data than the intercept only model.

Model Convergence Status
Convergence criterion (GCONV=1E-8) satisfied.

Testing Global Null Hypothesis: BETA=0			
**Test**	**Chi-Square**	**DF**	**Pr > ChiSq**
Likelihood Ratio	9989.6928	9	<.0001
Score	8564.9102	9	<.0001
Wald	6350.5204	9	<.0001

Overall model adequacy was assessed by global chi-square goodness of fit tests which showed the models as a whole fit significantly better than empty models.

Type 3 Analysis of Effects			
**Effect**	**DF**	**Wald Chi-Square**	**Pr > ChiSq**
gender	1	3974.4628	<.0001
child_gosr	1	328.4350	<.0001
ageatoffence	1	58.5092	<.0001
hmdc	1	56.0635	<.0001
eddc	1	2010.1415	<.0001
birth_cohort	1	10.0036	0.0016
prior_hmdc_alcohol	1	88.0228	<.0001
prior_dcp_notif	1	12.1499	0.0005
prior_courts	1	612.8732	<.0001

There are no commands in PROC LOGISTIC to check multicollinearity. Hence using a command like `corrb` will provide a correlation matrix for parameter estimator, then correlation coefficient which is large like > 0.8 can be dropped.

Estimated Covariance Matrix

Parameter	Intercept	gender0	child_gosr0	ageatoffence	hmdc1	eddc1	birth_cohort	prior_hmdc_alcohol0	prior_dcp_notif0	prior_courts0
Intercept	0.038183	-0.00104	-0.0005	-0.00128	-0.00051	-0.0004	-0.00356	-0.00261	-0.00064	-0.00102
gender0	-0.00104	0.001373	0.000368	8.095E-6	0.000079	0.000279	-0.00005	0.000167	0.000205	-0.00009
child_gosr0	-0.0005	0.000368	0.001367	1.921E-6	-0.00001	0.00008	2.299E-6	-0.00015	-0.00038	-0.00026
ageatoffence	-0.00128	8.095E-6	1.921E-6	0.00005	0.000019	-6.76E-6	0.000105	0.000041	-0.00002	0.000048
hmdc1	-0.00051	0.000079	-0.00001	0.000019	0.008393	-0.00155	-5.44E-6	0.000101	-0.00002	0.000065
eddc1	-0.0004	0.000279	0.00008	-6.76E-6	-0.00155	0.00202	-0.00004	0.000131	0.000027	0.000093
birth_cohort	-0.00356	-0.00005	2.299E-6	0.000105	-5.44E-6	-0.00004	0.000607	9.48E-6	0.000089	-4.76E-6
prior_hmdc_alcohol0	-0.00261	0.000167	-0.00015	0.000041	0.000101	0.000131	9.48E-6	0.002301	-0.00019	-0.00015
prior_dcp_notif0	-0.00064	0.000205	-0.00038	-0.00002	-0.00002	0.000027	0.000089	-0.00019	0.001686	-0.00017
prior_courts0	-0.00102	-0.00009	-0.00026	0.000048	0.000065	0.000093	-4.76E-6	-0.00015	-0.00017	0.001208

Estimated Correlation Matrix

Parameter	Intercept	gender0	child_gosr0	ageatoffence	hmdc1	eddc1	birth_cohort	prior_hmdc_alcohol0	prior_dcp_notif0	prior_courts0
Intercept	1.0000	-0.1442	-0.0690	-0.9314	-0.0283	-0.0460	-0.7392	-0.2783	-0.0795	-0.1502
gender0	-0.1442	1.0000	0.2687	0.0310	0.0232	0.1676	-0.0497	0.0941	0.1346	-0.0712

Estimated Correlation Matrix

Parameter	Intercept	gender0	child_gosr0	ageatoffence	hmdc1	eddc1	birth_cohort	prior_hmdc_alcohol0	prior_dcp_notif0	prior_courts0
child_gosr0	-0.0690	0.2687	1.0000	0.0074	-0.0035	0.0481	0.0025	-0.0864	-0.2494	-0.2044
ageatoffence	-0.9314	0.0310	0.0074	1.0000	0.0293	-0.0213	0.6029	0.1200	-0.0794	0.1962
hmdc1	-0.0283	0.0232	-0.0035	0.0293	1.0000	-0.3752	-0.0024	0.0230	-0.0050	0.0205
eddc1	-0.0460	0.1676	0.0481	-0.0213	-0.3752	1.0000	-0.0328	0.0607	0.0147	0.0593
birth_cohort	-0.7392	-0.0497	0.0025	0.6029	-0.0024	-0.0328	1.0000	0.0080	0.0882	-0.0056
prior_hmdc_alcohol0	-0.2783	0.0941	-0.0864	0.1200	0.0230	0.0607	0.0080	1.0000	-0.0942	-0.0910
prior_dcp_notif0	-0.0795	0.1346	-0.2494	-0.0794	-0.0050	0.0147	0.0882	-0.0942	1.0000	-0.1170
prior_courts0	-0.1502	-0.0712	-0.2044	0.1962	0.0205	0.0593	-0.0056	-0.0910	-0.1170	1.0000

A plot of predicted probabilities was performed to check the linearity assumption for the continuous variable AGE AT OFFENCE in the model:

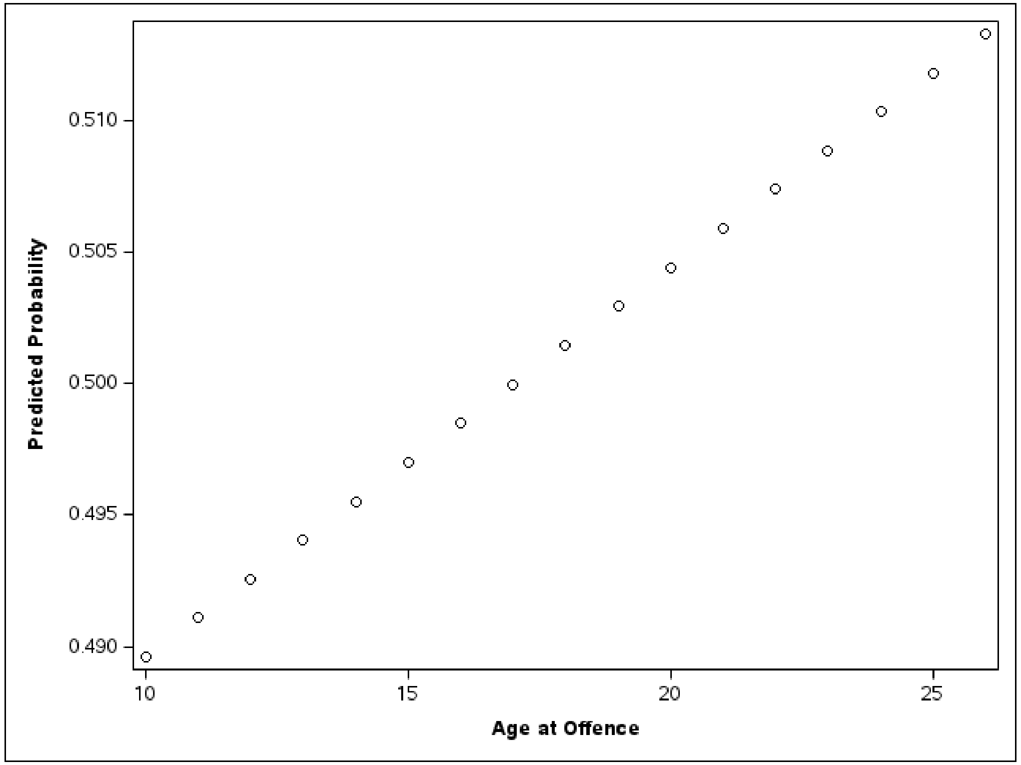

TABLE 3:

Model fit statistics for model in Table 3:

Model Fit Statistics		
**Criterion**	**Intercept Only**	**Intercept and Covariates**
AIC	1711.776	1650.765
SC	1716.980	1702.814
-2 Log L	1709.776	1630.765

Like the model in Table 2, the model for Table 3 that includes covariates is considered superior in terms of goodness of fit and model complexity. Lower values across all three criteria (AIC, BIC, and -2 Log L) for the model with covariates indicate that it provides a better representation of the data than the intercept only model.

**Model Convergence Status**
Convergence criterion (GCONV=1E-8) satisfied.

Testing Global Null Hypothesis: BETA=0			
**Test**	**Chi-Square**	**DF**	**Pr > ChiSq**
Likelihood Ratio	79.0110	9	<.0001
Score	80.6327	9	<.0001
Wald	74.1777	9	<.0001

Overall model adequacy was assessed by global chi-square goodness of fit tests which showed the models as a whole fit significantly better than empty models.

Type 3 Analysis of Effects

Effect	DF	Wald Chi-Square	Pr > ChiSq
gender	1	5.7526	0.0165
child_gosr	1	2.8189	0.0932
ageatoffence	1	0.8987	0.3431
eddc	1	2.8795	0.0897
birth_cohort	1	0.1654	0.6842
prior_hmdc_alcohol	1	10.5426	0.0012
prior_dcp_notif	1	0.4039	0.5251
prior_courts	1	2.2139	0.1368
victim	1	31.8439	<.0001

There are no commands in PROC LOGISTIC to check multicollinearity. Hence using a command like `corrb` will provide a correlation matrix for parameter estimator, then correlation coefficient which is large like > 0.8 can be dropped.

Estimated Covariance Matrix

Parameter	Intercept	gender0	child_gosr0	ageatoffence	eddc0	birth_cohort	prior_hmdc_alcohol0	prior_dcp_notif0	prior_courts0	victim1
Intercept	0.562117	-0.01095	-0.00912	-0.02019	-0.02304	-0.04567	-0.02539	-0.00252	-0.01106	-0.01098
gender0	-0.01095	0.021657	0.008447	-0.00002	-0.00238	-0.00028	0.001523	0.002802	-0.00197	-0.00304
child_gosr0	-0.00912	0.008447	0.022659	0.000033	-0.00355	-0.00031	-0.00246	-0.00711	-0.00418	0.002493
ageatoffence	-0.02019	-0.00002	0.000033	0.000848	0.000457	0.001321	0.000671	-0.00061	0.000593	-0.00039
eddc0	-0.02304	-0.00238	-0.00355	0.000457	0.236134	0.001966	0.004254	0.00095	0.00176	0.004343
birth_cohort	-0.04567	-0.00028	-0.00031	0.001321	0.001966	0.008067	-0.00056	0.001291	0.000102	-0.00007
prior_hmdc_alcohol0	-0.02539	0.001523	-0.00246	0.000671	0.004254	-0.00056	0.026164	-0.00403	-0.00125	-0.00411
prior_dcp_notif0	-0.00252	0.002802	-0.00711	-0.00061	0.00095	0.001291	-0.00403	0.025876	-0.00166	-0.00077
prior_courts0	-0.01106	-0.00197	-0.00418	0.000593	0.00176	0.000102	-0.00125	-0.00166	0.017101	-0.00299
victim1	-0.01098	-0.00304	0.002493	-0.00039	0.004343	-0.00007	-0.00411	-0.00077	-0.00299	0.028069

Estimated Correlation Matrix										
**Parameter**	**Intercept**	**gender0**	**child_gosr0**	**ageatoffence**	**eddc0**	**birth_cohort**	**prior_hmdc_alcohol0**	**prior_dcp_notif0**	**prior_courts0**	**victim1**
Intercept	1.0000	-0.0992	-0.0808	-0.9245	-0.0632	-0.6783	-0.2094	-0.0209	-0.1128	-0.0874
gender0	-0.0992	1.0000	0.3813	-0.0051	-0.0333	-0.0210	0.0640	0.1184	-0.1023	-0.1234
child_gosr0	-0.0808	0.3813	1.0000	0.0076	-0.0485	-0.0230	-0.1009	-0.2937	-0.2123	0.0988
ageatoffence	-0.9245	-0.0051	0.0076	1.0000	0.0323	0.5052	0.1426	-0.1306	0.1558	-0.0789
eddc0	-0.0632	-0.0333	-0.0485	0.0323	1.0000	0.0451	0.0541	0.0122	0.0277	0.0533
birth_cohort	-0.6783	-0.0210	-0.0230	0.5052	0.0451	1.0000	-0.0387	0.0894	0.0087	-0.0048
prior_hmdc_alcohol0	-0.2094	0.0640	-0.1009	0.1426	0.0541	-0.0387	1.0000	-0.1549	-0.0593	-0.1518
prior_dcp_notif0	-0.0209	0.1184	-0.2937	-0.1306	0.0122	0.0894	-0.1549	1.0000	-0.0790	-0.0287
prior_courts0	-0.1128	-0.1023	-0.2123	0.1558	0.0277	0.0087	-0.0593	-0.0790	1.0000	-0.1365
victim1	-0.0874	-0.1234	0.0988	-0.0789	0.0533	-0.0048	-0.1518	-0.0287	-0.1365	1.0000

A plot of predicted probabilities was performed to check the linearity assumption for the continuous variable AGE AT OFFENCE in the model:

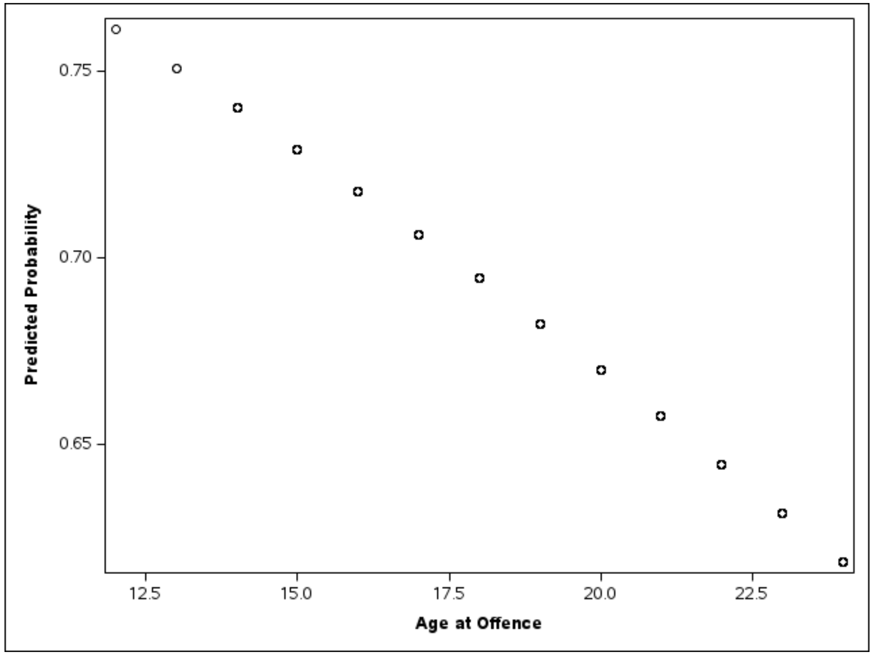

FIGURE 1:

Model fit statistics using GEE Fit Criteria:

Offence type risk factor	Emergency department model QIC	Hospital Admissions model QIC
Assault causing bodily harm	34928.7	10842.5
Common assault	35599.2	10511.8
Wounding	35452.6	10786.1
Grievous bodily harm	35952.2	10569.8
Dangerous driving causing grievous bodily harm	35850.2	10591.7
Damage	35818.4	10848.5
Criminal damage	35939.4	10852.6
Deprivation of liberty	36058.3	10915.6
Act intended to cause grievous bodily harm or prevent arrest	36050.4	10889.5
Stealing	35821.6	10884.4
Robbery in circumstances of aggravation	36042.6	10896.0
Dangerous driving causing death	36042.9	10858.7
Breach violence restraining order	35971.3	10904.1
Threats to harm, take control of conveyance or building	36029.6	10902.3
Burglary and commit	36013.4	10899.1
Breach police restraining order	35984.2	10876.4
Robbery	36058.8	10903.1
Assault serious	36066.7	10917.8
Going armed in public to cause fear	36056.0	10911.4
Act or omission with intent to harm and causing bodily harm	36058.1	10907.4

The Quasi-Likelihood under Independence Model Criterion (QIC) used to assess model fit also indicated the models with covariates provided a better representation of the data than the intercept only model. Overall model adequacy showed the models as a whole fit significantly better than empty models.

In SAS PROC GENMOD, there isn't a direct option to assess collinearity (e.g. `COLLIN`).

Like the options used for the PROC LOGISTIC models, the `CORRB` option was used to obtain the correlation or covariance matrix of parameter estimates, which can be relevant to assessing multicollinearity.

Note that no continuous variables were used in the RR models and hence linearity assumption testing was not required.

Emergency department presentation relative risk models:

Assault causing bodily harm

Parameter Information

Parameter	Effect	gender	child_gosr	seifa5	ra3	birth_cohort	assault_harm
Prm1	Intercept						
Prm2	gender	1					
Prm3	gender	0					
Prm4	child_gosr		1				
Prm5	child_gosr		0				
Prm6	seifa5			2			
Prm7	seifa5			3			
Prm8	seifa5			4			
Prm9	seifa5			5			
Prm10	seifa5			1			
Prm11	ra3				2		
Prm12	ra3				3		
Prm13	ra3				1		
Prm14	birth_cohort					2	
Prm15	birth_cohort					3	
Prm16	birth_cohort					4	
Prm17	birth_cohort					1	
Prm18	assault_harm						1
Prm19	assault_harm						0

Estimated Correlation Matrix

	Prm1	Prm2	Prm4	Prm6	Prm7	Prm8	Prm9	Prm11	Prm12	Prm14	Prm15	Prm16	Prm18
Prm1	1.0000	-0.3989	-0.2582	-0.3205	-0.2975	-0.3103	-0.3310	-0.3304	-0.2316	-0.5971	-0.5812	-0.3913	-0.1813

	Estimated Correlation Matrix												
	**Prm1**	**Prm2**	**Prm4**	**Prm6**	**Prm7**	**Prm8**	**Prm9**	**Prm11**	**Prm12**	**Prm14**	**Prm15**	**Prm16**	**Prm18**
Prm2	-0.3989	1.0000	0.1175	0.0042	-0.0147	-0.0201	-0.0147	0.0125	0.0390	0.0408	0.0689	0.0675	-0.1067
Prm4	-0.2582	0.1175	1.0000	0.1138	0.1639	0.1779	0.1808	-0.0738	-0.4866	0.0053	-0.0045	-0.0195	-0.0414
Prm6	-0.3205	0.0042	0.1138	1.0000	0.3325	0.3111	0.2864	0.1113	0.0163	0.0028	-0.0153	-0.0043	-0.0189
Prm7	-0.2975	-0.0147	0.1639	0.3325	1.0000	0.2834	0.2670	0.0808	0.0357	0.0009	0.0008	0.0115	-0.0176
Prm8	-0.3103	-0.0201	0.1779	0.3111	0.2834	1.0000	0.2785	0.1677	0.0933	0.0026	-0.0046	-0.0007	-0.0225
Prm9	-0.3310	-0.0147	0.1808	0.2864	0.2670	0.2785	1.0000	0.2489	0.1640	-0.0023	0.0020	0.0127	-0.0130
Prm11	-0.3304	0.0125	-0.0738	0.1113	0.0808	0.1677	0.2489	1.0000	0.4116	0.0057	-0.0016	0.0047	0.0222
Prm12	-0.2316	0.0390	-0.4866	0.0163	0.0357	0.0933	0.1640	0.4116	1.0000	0.0117	0.0209	0.0175	0.0007
Prm14	-0.5971	0.0408	0.0053	0.0028	0.0009	0.0026	-0.0023	0.0057	0.0117	1.0000	0.7036	0.4602	-0.0264
Prm15	-0.5812	0.0689	-0.0045	-0.0153	0.0008	-0.0046	0.0020	-0.0016	0.0209	0.7036	1.0000	0.4425	-0.0081
Prm16	-0.3913	0.0675	-0.0195	-0.0043	0.0115	-0.0007	0.0127	0.0047	0.0175	0.4602	0.4425	1.0000	0.0036
Prm18	-0.1813	-0.1067	-0.0414	-0.0189	-0.0176	-0.0225	-0.0130	0.0222	0.0007	-0.0264	-0.0081	0.0036	1.0000

Common assault

		Parameter Information					
**Parameter**	**Effect**	**gender**	**child_gosr**	**seifa5**	**ra3**	**birth_cohort**	**common_assault**
Prm1	Intercept						
Prm2	gender	1					
Prm3	gender	0					
Prm4	child_gosr		1				
Prm5	child_gosr		0				
Prm6	seifa5			2			
Prm7	seifa5			3			
Prm8	seifa5			4			
Prm9	seifa5			5			
Prm10	seifa5			1			
Prm11	ra3				2		
Prm12	ra3				3		
Prm13	ra3				1		
Prm14	birth_cohort					2	
Prm15	birth_cohort					3	
Prm16	birth_cohort					4	

Parameter Information

Parameter	Effect	gender	child_gosr	seifa5	ra3	birth_cohort	common_assault
Prm17	birth_cohort					1	
Prm18	common_assault						1
Prm19	common_assault						0

Estimated Correlation Matrix

	Prm1	Prm2	Prm4	Prm6	Prm7	Prm8	Prm9	Prm11	Prm12	Prm14	Prm15	Prm16	Prm18
Prm1	1.0000	-0.4269	-0.2592	-0.3281	-0.3066	-0.3201	-0.3395	-0.3290	-0.2373	-0.5989	-0.5787	-0.3854	-0.1901
Prm2	-0.4269	1.0000	0.1022	0.0080	-0.0134	-0.0206	-0.0086	0.0232	0.0393	0.0337	0.0606	0.0596	0.0668
Prm4	-0.2592	0.1022	1.0000	0.1122	0.1623	0.1794	0.1809	-0.0756	-0.4796	0.0024	-0.0119	-0.0187	0.0011
Prm6	-0.3281	0.0080	0.1122	1.0000	0.3322	0.3104	0.2857	0.1117	0.0175	0.0029	-0.0137	-0.0049	0.0108
Prm7	-0.3066	-0.0134	0.1623	0.3322	1.0000	0.2839	0.2675	0.0851	0.0394	0.0028	0.0042	0.0149	0.0097
Prm8	-0.3201	-0.0206	0.1794	0.3104	0.2839	1.0000	0.2792	0.1673	0.0970	0.0054	-0.0026	0.0038	0.0103
Prm9	-0.3395	-0.0086	0.1809	0.2857	0.2675	0.2792	1.0000	0.2511	0.1696	-0.0032	0.0045	0.0121	0.0101
Prm11	-0.3290	0.0232	-0.0756	0.1117	0.0851	0.1673	0.2511	1.0000	0.4157	0.0075	0.0023	0.0002	-0.0113
Prm12	-0.2373	0.0393	-0.4796	0.0175	0.0394	0.0970	0.1696	0.4157	1.0000	0.0110	0.0253	0.0120	0.0055
Prm14	-0.5989	0.0337	0.0024	0.0029	0.0028	0.0054	-0.0032	0.0075	0.0110	1.0000	0.7036	0.4602	-0.0092
Prm15	-0.5787	0.0606	-0.0119	-0.0137	0.0042	-0.0026	0.0045	0.0023	0.0253	0.7036	1.0000	0.4422	-0.0107
Prm16	-0.3854	0.0596	-0.0187	-0.0049	0.0149	0.0038	0.0121	0.0002	0.0120	0.4602	0.4422	1.0000	-0.0046
Prm18	-0.1901	0.0668	0.0011	0.0108	0.0097	0.0103	0.0101	-0.0113	0.0055	-0.0092	-0.0107	-0.0046	1.0000

Wounding

Parameter Information

Parameter	Effect	gender	child_gosr	seifa5	ra3	birth_cohort	wound
Prm1	Intercept						
Prm2	gender	1					
Prm3	gender	0					
Prm4	child_gosr		1				
Prm5	child_gosr		0				
Prm6	seifa5			2			
Prm7	seifa5			3			

Parameter Information

Parameter	Effect	gender	child_gos	rseifa5	ra3	birth_cohort	wound
Prm8	seifa5			4			
Prm9	seifa5			5			
Prm10	seifa5			1			
Prm11	ra3				2		
Prm12	ra3				3		
Prm13	ra3				1		
Prm14	birth_cohort					2	
Prm15	birth_cohort					3	
Prm16	birth_cohort					4	
Prm17	birth_cohort					1	
Prm18	wound						1
Prm19	wound						0

Estimated Correlation Matrix

	Prm1	Prm2	Prm4	Prm6	Prm7	Prm8	Prm9	Prm11	Prm12	Prm14	Prm15	Prm16	Prm18
Prm1	1.0000	-0.4208	-0.2527	-0.3308	-0.3062	-0.3235	-0.3394	-0.3352	-0.2393	-0.6137	-0.5919	-0.3958	-0.0667
Prm2	-0.4208	1.0000	0.1101	0.0086	-0.0127	-0.0204	-0.0040	0.0202	0.0408	0.0369	0.0675	0.0650	-0.0627
Prm4	-0.2527	0.1101	1.0000	0.1109	0.1592	0.1767	0.1781	-0.0798	-0.4866	0.0006	-0.0108	-0.0216	-0.0878
Prm6	-0.3308	0.0086	0.1109	1.0000	0.3314	0.3099	0.2856	0.1142	0.0165	0.0062	-0.0125	-0.0039	-0.0144
Prm7	-0.3062	-0.0127	0.1592	0.3314	1.0000	0.2827	0.2661	0.0821	0.0386	0.0024	0.0034	0.0168	-0.0118
Prm8	-0.3235	-0.0204	0.1767	0.3099	0.2827	1.0000	0.2777	0.1645	0.0952	0.0120	0.0007	0.0057	-0.0111
Prm9	-0.3394	-0.0040	0.1781	0.2856	0.2661	0.2777	1.0000	0.2506	0.1678	-0.0049	0.0001	0.0145	-0.0180
Prm11	-0.3352	0.0202	-0.0798	0.1142	0.0821	0.1645	0.2506	1.0000	0.4138	0.0067	0.0025	0.0015	0.0193
Prm12	-0.2393	0.0408	-0.4866	0.0165	0.0386	0.0952	0.1678	0.4138	1.0000	0.0171	0.0317	0.0200	-0.0228
Prm14	-0.6137	0.0369	0.0006	0.0062	0.0024	0.0120	-0.0049	0.0067	0.0171	1.0000	0.7035	0.4602	0.0140
Prm15	-0.5919	0.0675	-0.0108	-0.0125	0.0034	0.0007	0.0001	0.0025	0.0317	0.7035	1.0000	0.4425	-0.0104
Prm16	-0.3958	0.0650	-0.0216	-0.0039	0.0168	0.0057	0.0145	0.0015	0.0200	0.4602	0.4425	1.0000	-0.0004
Prm18	-0.0667	-0.0627	-0.0878	-0.0144	-0.0118	-0.0111	-0.0180	0.0193	-0.0228	0.0140	-0.0104	-0.0004	1.0000

Grievous bodily harm

Parameter Information

Parameter	Effect	gender	child_gos	rseifa5	ra3	birth_cohort	grievous
Prm1	Intercept						

Parameter Information

Parameter	Effect	gender	child_gosr	seifa5	ra3	birth_cohort	grievous
Prm2	gender	1					
Prm3	gender	0					
Prm4	child_gosr		1				
Prm5	child_gosr		0				
Prm6	seifa5			2			
Prm7	seifa5			3			
Prm8	seifa5			4			
Prm9	seifa5			5			
Prm10	seifa5			1			
Prm11	ra3				2		
Prm12	ra3				3		
Prm13	ra3				1		
Prm14	birth_cohort					2	
Prm15	birth_cohort					3	
Prm16	birth_cohort					4	
Prm17	birth_cohort					1	
Prm18	grievous						1
Prm19	grievous						0

Estimated Correlation Matrix

	Prm1	Prm2	Prm4	Prm6	Prm7	Prm8	Prm9	Prm11	Prm12	Prm14	Prm15	Prm16	Prm18
Prm1	1.0000	-0.4183	-0.2633	-0.3316	-0.3080	-0.3211	-0.3416	-0.3362	-0.2403	-0.6133	-0.5928	-0.3961	-0.0037
Prm2	-0.4183	1.0000	0.1026	0.0066	-0.0158	-0.0210	-0.0095	0.0157	0.0331	0.0374	0.0664	0.0646	-0.1131
Prm4	-0.2633	0.1026	1.0000	0.1125	0.1621	0.1765	0.1796	-0.0741	-0.4841	0.0023	-0.0127	-0.0207	0.0063
Prm6	-0.3316	0.0066	0.1125	1.0000	0.3320	0.3105	0.2861	0.1133	0.0152	0.0039	-0.0144	-0.0008	-0.0118
Prm7	-0.3080	-0.0158	0.1621	0.3320	1.0000	0.2827	0.2663	0.0830	0.0358	0.0034	0.0029	0.0136	-0.0002
Prm8	-0.3211	-0.0210	0.1765	0.3105	0.2827	1.0000	0.2785	0.1672	0.0942	0.0052	-0.0030	0.0037	-0.0185
Prm9	-0.3416	-0.0095	0.1796	0.2861	0.2663	0.2785	1.0000	0.2511	0.1673	-0.0024	0.0032	0.0150	-0.0267
Prm11	-0.3362	0.0157	-0.0741	0.1133	0.0830	0.1672	0.2511	1.0000	0.4137	0.0073	0.0028	0.0027	0.0219
Prm12	-0.2403	0.0331	-0.4841	0.0152	0.0358	0.0942	0.1673	0.4137	1.0000	0.0148	0.0314	0.0169	0.0160
Prm14	-0.6133	0.0374	0.0023	0.0039	0.0034	0.0052	-0.0024	0.0073	0.0148	1.0000	0.7038	0.4605	-0.0215
Prm15	-0.5928	0.0664	-0.0127	-0.0144	0.0029	-0.0030	0.0032	0.0028	0.0314	0.7038	1.0000	0.4426	-0.0348
Prm16	-0.3961	0.0646	-0.0207	-0.0008	0.0136	0.0037	0.0150	0.0027	0.0169	0.4605	0.4426	1.0000	-0.0238
Prm18	-0.0037	-0.1131	0.0063	-0.0118	-0.0002	-0.0185	-0.0267	0.0219	0.0160	-0.0215	-0.0348	-0.0238	1.0000

Dangerous driving causing grievous bodily harm

Parameter Information

Parameter	Effect	gender	child_gosr	seifa5	ra3	birth_cohort	danger_harm
Prm1	Intercept						
Prm2	gender	1					
Prm3	gender	0					
Prm4	child_gosr		1				
Prm5	child_gosr		0				
Prm6	seifa5			2			
Prm7	seifa5			3			
Prm8	seifa5			4			
Prm9	seifa5			5			
Prm10	seifa5			1			
Prm11	ra3				2		
Prm12	ra3				3		
Prm13	ra3				1		
Prm14	birth_cohort					2	
Prm15	birth_cohort					3	
Prm16	birth_cohort					4	
Prm17	birth_cohort					1	
Prm18	danger_harm						1
Prm19	danger_harm						0

Estimated Correlation Matrix

	Prm1	Prm2	Prm4	Prm6	Prm7	Prm8	Prm9	Prm11	Prm12	Prm14	Prm15	Prm16	Prm18
Prm1	1.0000	-0.4203	-0.2673	-0.3317	-0.3094	-0.3236	-0.3442	-0.3338	-0.2378	-0.6135	-0.5936	-0.3923	-0.0035
Prm2	-0.4203	1.0000	0.1018	0.0058	-0.0146	-0.0178	-0.0087	0.0186	0.0327	0.0343	0.0661	0.0639	-0.0333
Prm4	-0.2673	0.1018	1.0000	0.1123	0.1619	0.1773	0.1802	-0.0742	-0.4821	0.0044	-0.0101	-0.0222	0.0613
Prm6	-0.3317	0.0058	0.1123	1.0000	0.3320	0.3107	0.2858	0.1125	0.0162	0.0050	-0.0130	-0.0023	-0.0178
Prm7	-0.3094	-0.0146	0.1619	0.3320	1.0000	0.2829	0.2668	0.0852	0.0376	0.0027	0.0042	0.0128	0.0042
Prm8	-0.3236	-0.0178	0.1773	0.3107	0.2829	1.0000	0.2784	0.1668	0.0971	0.0071	-0.0021	0.0065	-0.0376
Prm9	-0.3442	-0.0087	0.1802	0.2858	0.2668	0.2784	1.0000	0.2524	0.1686	-0.0019	0.0047	0.0158	-0.0072
Prm11	-0.3338	0.0186	-0.0742	0.1125	0.0852	0.1668	0.2524	1.0000	0.4138	0.0080	0.0039	0.0018	-0.0517
Prm12	-0.2378	0.0327	-0.4821	0.0162	0.0376	0.0971	0.1686	0.4138	1.0000	0.0147	0.0304	0.0159	-0.0386
Prm14	-0.6135	0.0343	0.0044	0.0050	0.0027	0.0071	-0.0019	0.0080	0.0147	1.0000	0.7033	0.4594	-0.0134
Prm15	-0.5936	0.0661	-0.0101	-0.0130	0.0042	-0.0021	0.0047	0.0039	0.0304	0.7033	1.0000	0.4439	-0.0448

Estimated Correlation Matrix

	Prm1	Prm2	Prm4	Prm6	Prm7	Prm8	Prm9	Prm11	Prm12	Prm14	Prm15	Prm16	Prm18
Prm16	-0.3923	0.0639	-0.0222	-0.0023	0.0128	0.0065	0.0158	0.0018	0.0159	0.4594	0.4439	1.0000	-0.0861
Prm18	-0.0035	-0.0333	0.0613	-0.0178	0.0042	-0.0376	-0.0072	-0.0517	-0.0386	-0.0134	-0.0448	-0.0861	1.0000

Damage

Parameter Information

Parameter	Effect	gender	child_gosr	seifa5	ra3	birth_cohort	damage
Prm1	Intercept						
Prm2	gender	1					
Prm3	gender	0					
Prm4	child_gosr		1				
Prm5	child_gosr		0				
Prm6	seifa5			2			
Prm7	seifa5			3			
Prm8	seifa5			4			
Prm9	seifa5			5			
Prm10	seifa5			1			
Prm11	ra3				2		
Prm12	ra3				3		
Prm13	ra3				1		
Prm14	birth_cohort					2	
Prm15	birth_cohort					3	
Prm16	birth_cohort					4	
Prm17	birth_cohort					1	
Prm18	damage						1
Prm19	damage						0

Estimated Correlation Matrix

	Prm1	Prm2	Prm4	Prm6	Prm7	Prm8	Prm9	Prm11	Prm12	Prm14	Prm15	Prm16	Prm18
Prm1	1.0000	-0.4235	-0.2639	-0.3302	-0.3059	-0.3226	-0.3416	-0.3342	-0.2397	-0.6130	-0.5951	-0.3991	-0.0739
Prm2	-0.4235	1.0000	0.1048	0.0057	-0.0159	-0.0210	-0.0108	0.0167	0.0341	0.0372	0.0685	0.0670	0.0157
Prm4	-0.2639	0.1048	1.0000	0.1111	0.1589	0.1762	0.1794	-0.0737	-0.4849	0.0017	-0.0101	-0.0189	0.0322
Prm6	-0.3302	0.0057	0.1111	1.0000	0.3312	0.3094	0.2847	0.1116	0.0140	0.0053	-0.0128	-0.0012	0.0016
Prm7	-0.3059	-0.0159	0.1589	0.3312	1.0000	0.2822	0.2658	0.0826	0.0377	0.0030	0.0030	0.0141	-0.0006

Estimated Correlation Matrix													
	**Prm1**	**Prm2**	**Prm4**	**Prm6**	**Prm7**	**Prm8**	**Prm9**	**Prm11**	**Prm12**	**Prm14**	**Prm15**	**Prm16**	**Prm18**
Prm8	-0.3226	-0.0210	0.1762	0.3094	0.2822	1.0000	0.2780	0.1673	0.0962	0.0071	-0.0016	0.0041	0.0072
Prm9	-0.3416	-0.0108	0.1794	0.2847	0.2658	0.2780	1.0000	0.2516	0.1677	-0.0028	0.0040	0.0153	0.0108
Prm11	-0.3342	0.0167	-0.0737	0.1116	0.0826	0.1673	0.2516	1.0000	0.4127	0.0100	0.0039	0.0036	-0.0095
Prm12	-0.2397	0.0341	-0.4849	0.0140	0.0377	0.0962	0.1677	0.4127	1.0000	0.0165	0.0308	0.0195	0.0109
Prm14	-0.6130	0.0372	0.0017	0.0053	0.0030	0.0071	-0.0028	0.0100	0.0165	1.0000	0.7036	0.4603	0.0032
Prm15	-0.5951	0.0685	-0.0101	-0.0128	0.0030	-0.0016	0.0040	0.0039	0.0308	0.7036	1.0000	0.4428	0.0179
Prm16	-0.3991	0.0670	-0.0189	-0.0012	0.0141	0.0041	0.0153	0.0036	0.0195	0.4603	0.4428	1.0000	0.0235
Prm18	-0.0739	0.0157	0.0322	0.0016	-0.0006	0.0072	0.0108	-0.0095	0.0109	0.0032	0.0179	0.0235	1.0000

Criminal damage

Parameter Information							
**Parameter**	**Effect**	**gender**	**child_gosr**	**seifa5**	**ra3**	**birth_cohort**	**crim_damage**
Prm1	Intercept						
Prm2	gender	1					
Prm3	gender	0					
Prm4	child_gosr		1				
Prm5	child_gosr		0				
Prm6	seifa5			2			
Prm7	seifa5			3			
Prm8	seifa5			4			
Prm9	seifa5			5			
Prm10	seifa5			1			
Prm11	ra3				2		
Prm12	ra3				3		
Prm13	ra3				1		
Prm14	birth_cohort					2	
Prm15	birth_cohort					3	
Prm16	birth_cohort					4	
Prm17	birth_cohort					1	
Prm18	crim_damage						1
Prm19	crim_damage						0

Estimated Correlation Matrix													
	**Prm1**	**Prm2**	**Prm4**	**Prm6**	**Prm7**	**Prm8**	**Prm9**	**Prm11**	**Prm12**	**Prm14**	**Prm15**	**Prm16**	**Prm18**
Prm1	1.0000	-0.4228	-0.2639	-0.3305	-0.3080	-0.3211	-0.3421	-0.3345	-0.2413	-0.6127	-0.5935	-0.3979	-0.0710
Prm2	-0.4228	1.0000	0.1030	0.0048	-0.0148	-0.0215	-0.0101	0.0186	0.0356	0.0375	0.0669	0.0665	0.0038
Prm4	-0.2639	0.1030	1.0000	0.1123	0.1619	0.1773	0.1807	-0.0732	-0.4835	0.0026	-0.0102	-0.0198	0.0140
Prm6	-0.3305	0.0048	0.1123	1.0000	0.3319	0.3101	0.2856	0.1141	0.0145	0.0046	-0.0141	-0.0024	-0.0020
Prm7	-0.3080	-0.0148	0.1619	0.3319	1.0000	0.2830	0.2667	0.0843	0.0371	0.0027	0.0027	0.0141	0.0093
Prm8	-0.3211	-0.0215	0.1773	0.3101	0.2830	1.0000	0.2786	0.1678	0.0966	0.0034	-0.0044	0.0021	0.0079
Prm9	-0.3421	-0.0101	0.1807	0.2856	0.2667	0.2786	1.0000	0.2517	0.1677	-0.0040	0.0023	0.0134	0.0173
Prm11	-0.3345	0.0186	-0.0732	0.1141	0.0843	0.1678	0.2517	1.0000	0.4131	0.0064	0.0006	0.0023	0.0064
Prm12	-0.2413	0.0356	-0.4835	0.0145	0.0371	0.0966	0.1677	0.4131	1.0000	0.0158	0.0306	0.0196	0.0276
Prm14	-0.6127	0.0375	0.0026	0.0046	0.0027	0.0034	-0.0040	0.0064	0.0158	1.0000	0.7037	0.4605	0.0108
Prm15	-0.5935	0.0669	-0.0102	-0.0141	0.0027	-0.0044	0.0023	0.0006	0.0306	0.7037	1.0000	0.4427	0.0175
Prm16	-0.3979	0.0665	-0.0198	-0.0024	0.0141	0.0021	0.0134	0.0023	0.0196	0.4605	0.4427	1.0000	0.0199
Prm18	-0.0710	0.0038	0.0140	-0.0020	0.0093	0.0079	0.0173	0.0064	0.0276	0.0108	0.0175	0.0199	1.0000

Deprivation of liberty

Parameter Information							
**Parameter**	**Effect**	**gender**	**child_gosr**	**seifa5**	**ra3**	**birth_cohort**	**deprivation**
Prm1	Intercept						
Prm2	gender	1					
Prm3	gender	0					
Prm4	child_gosr		1				
Prm5	child_gosr		0				
Prm6	seifa5			2			
Prm7	seifa5			3			
Prm8	seifa5			4			
Prm9	seifa5			5			
Prm10	seifa5			1			
Prm11	ra3				2		
Prm12	ra3				3		
Prm13	ra3				1		
Prm14	birth_cohort					2	
Prm15	birth_cohort					3	
Prm16	birth_cohort					4	
Prm17	birth_cohort					1	

Parameter Information

Parameter	Effect	gender	child_gosr	seifa5	ra3	birth_cohort	deprivation
Prm18	deprivation						1
Prm19	deprivation						0

Estimated Correlation Matrix

	Prm1	Prm2	Prm4	Prm6	Prm7	Prm8	Prm9	Prm11	Prm12	Prm14	Prm15	Prm16	Prm18
Prm1	1.0000	-0.4220	-0.2618	-0.3312	-0.3078	-0.3217	-0.3422	-0.3355	-0.2401	-0.6139	-0.5943	-0.3976	-0.0655
Prm2	-0.4220	1.0000	0.1011	0.0056	-0.0152	-0.0226	-0.0101	0.0183	0.0344	0.0364	0.0657	0.0643	0.0238
Prm4	-0.2618	0.1011	1.0000	0.1117	0.1616	0.1767	0.1798	-0.0730	-0.4823	0.0029	-0.0119	-0.0202	-0.0111
Prm6	-0.3312	0.0056	0.1117	1.0000	0.3319	0.3100	0.2856	0.1146	0.0158	0.0044	-0.0144	-0.0010	-0.0044
Prm7	-0.3078	-0.0152	0.1616	0.3319	1.0000	0.2829	0.2665	0.0850	0.0370	0.0029	0.0025	0.0137	0.0022
Prm8	-0.3217	-0.0226	0.1767	0.3100	0.2829	1.0000	0.2781	0.1670	0.0965	0.0057	-0.0033	0.0036	0.0072
Prm9	-0.3422	-0.0101	0.1798	0.2856	0.2665	0.2781	1.0000	0.2521	0.1684	-0.0035	0.0026	0.0139	0.0122
Prm11	-0.3355	0.0183	-0.0730	0.1146	0.0850	0.1670	0.2521	1.0000	0.4132	0.0071	0.0017	0.0030	0.0086
Prm12	-0.2401	0.0344	-0.4823	0.0158	0.0370	0.0965	0.1684	0.4132	1.0000	0.0150	0.0301	0.0172	0.0079
Prm14	-0.6139	0.0364	0.0029	0.0044	0.0029	0.0057	-0.0035	0.0071	0.0150	1.0000	0.7038	0.4606	0.0247
Prm15	-0.5943	0.0657	-0.0119	-0.0144	0.0025	-0.0033	0.0026	0.0017	0.0301	0.7038	1.0000	0.4428	0.0393
Prm16	-0.3976	0.0643	-0.0202	-0.0010	0.0137	0.0036	0.0139	0.0030	0.0172	0.4606	0.4428	1.0000	0.0259
Prm18	-0.0655	0.0238	-0.0111	-0.0044	0.0022	0.0072	0.0122	0.0086	0.0079	0.0247	0.0393	0.0259	1.0000

Act intended to cause grievous bodily harm or prevent arrest

Parameter Information

Parameter	Effect	gender	child_gosr	seifa5	ra3	birth_cohort	act_arrest
Prm1	Intercept						
Prm2	gender	1					
Prm3	gender	0					
Prm4	child_gosr		1				
Prm5	child_gosr		0				
Prm6	seifa5			2			
Prm7	seifa5			3			
Prm8	seifa5			4			
Prm9	seifa5			5			
Prm10	seifa5			1			
Prm11	ra3				2		

Parameter	Effect	gender	child_gosr	seifa5	ra3	birth_cohort	act_arrest
**Parameter Information**							
Prm12	ra3				3		
Prm13	ra3				1		
Prm14	birth_cohort					2	
Prm15	birth_cohort					3	
Prm16	birth_cohort					4	
Prm17	birth_cohort					1	
Prm18	act_arrest						1
Prm19	act_arrest						0

Estimated Correlation Matrix

	Prm1	Prm2	Prm4	Prm6	Prm7	Prm8	Prm9	Prm11	Prm12	Prm14	Prm15	Prm16	Prm18
Prm1	1.0000	-0.4210	-0.2632	-0.3317	-0.3080	-0.3221	-0.3422	-0.3365	-0.2408	-0.6134	-0.5933	-0.3965	-0.0061
Prm2	-0.4210	1.0000	0.1023	0.0059	-0.0152	-0.0222	-0.0100	0.0174	0.0335	0.0358	0.0651	0.0635	-0.0441
Prm4	-0.2632	0.1023	1.0000	0.1119	0.1619	0.1779	0.1801	-0.0733	-0.4820	0.0032	-0.0117	-0.0205	-0.0133
Prm6	-0.3317	0.0059	0.1119	1.0000	0.3320	0.3101	0.2856	0.1142	0.0153	0.0042	-0.0146	-0.0016	-0.0054
Prm7	-0.3080	-0.0152	0.1619	0.3320	1.0000	0.2831	0.2666	0.0851	0.0369	0.0021	0.0021	0.0132	-0.0005
Prm8	-0.3221	-0.0222	0.1779	0.3101	0.2831	1.0000	0.2783	0.1665	0.0955	0.0055	-0.0029	0.0032	-0.0134
Prm9	-0.3422	-0.0100	0.1801	0.2856	0.2666	0.2783	1.0000	0.2517	0.1681	-0.0039	0.0027	0.0134	-0.0041
Prm11	-0.3365	0.0174	-0.0733	0.1142	0.0851	0.1665	0.2517	1.0000	0.4141	0.0071	0.0020	0.0027	0.0378
Prm12	-0.2408	0.0335	-0.4820	0.0153	0.0369	0.0955	0.1681	0.4141	1.0000	0.0147	0.0304	0.0176	0.0334
Prm14	-0.6134	0.0358	0.0032	0.0042	0.0021	0.0055	-0.0039	0.0071	0.0147	1.0000	0.7036	0.4601	-0.0112
Prm15	-0.5933	0.0651	-0.0117	-0.0146	0.0021	-0.0029	0.0027	0.0020	0.0304	0.7036	1.0000	0.4422	-0.0153
Prm16	-0.3965	0.0635	-0.0205	-0.0016	0.0132	0.0032	0.0134	0.0027	0.0176	0.4601	0.4422	1.0000	0.0049
Prm18	-0.0061	-0.0441	-0.0133	-0.0054	-0.0005	-0.0134	-0.0041	0.0378	0.0334	-0.0112	-0.0153	0.0049	1.0000

Stealing

Parameter Information

Parameter	Effect	gender	child_gosr	seifa5	ra3	birth_cohort	steal
Prm1	Intercept						
Prm2	gender	1					
Prm3	gender	0					
Prm4	child_gosr		1				

Parameter Information							
Parameter	Effect	gender	child_gosr	seifa5	ra3	birth_cohort	steal
Prm5	child_gosr		0				
Prm6	seifa5			2			
Prm7	seifa5			3			
Prm8	seifa5			4			
Prm9	seifa5			5			
Prm10	seifa5			1			
Prm11	ra3				2		
Prm12	ra3				3		
Prm13	ra3				1		
Prm14	birth_cohort					2	
Prm15	birth_cohort					3	
Prm16	birth_cohort					4	
Prm17	birth_cohort					1	
Prm18	steal						1
Prm19	steal						0

Estimated Correlation Matrix													
	Prm1	Prm2	Prm4	Prm6	Prm7	Prm8	Prm9	Prm11	Prm12	Prm14	Prm15	Prm16	Prm18
Prm1	1.0000	-0.4241	-0.2661	-0.3315	-0.3062	-0.3199	-0.3406	-0.3336	-0.2367	-0.6142	-0.5936	-0.3960	-0.0433
Prm2	-0.4241	1.0000	0.1066	0.0061	-0.0156	-0.0250	-0.0123	0.0163	0.0332	0.0385	0.0686	0.0658	0.0042
Prm4	-0.2661	0.1066	1.0000	0.1110	0.1607	0.1758	0.1778	-0.0745	-0.4868	0.0062	-0.0083	-0.0175	0.0190
Prm6	-0.3315	0.0061	0.1110	1.0000	0.3318	0.3101	0.2857	0.1158	0.0161	0.0044	-0.0153	-0.0021	0.0023
Prm7	-0.3062	-0.0156	0.1607	0.3318	1.0000	0.2824	0.2658	0.0839	0.0347	0.0029	0.0028	0.0119	-0.0068
Prm8	-0.3199	-0.0250	0.1758	0.3101	0.2824	1.0000	0.2777	0.1662	0.0950	0.0054	-0.0036	0.0033	0.0022
Prm9	-0.3406	-0.0123	0.1778	0.2857	0.2658	0.2777	1.0000	0.2517	0.1672	-0.0027	0.0038	0.0153	-0.0077
Prm11	-0.3336	0.0163	-0.0745	0.1158	0.0839	0.1662	0.2517	1.0000	0.4129	0.0064	0.0008	0.0009	0.0013
Prm12	-0.2367	0.0332	-0.4868	0.0161	0.0347	0.0950	0.1672	0.4129	1.0000	0.0133	0.0279	0.0159	0.0087
Prm14	-0.6142	0.0385	0.0062	0.0044	0.0029	0.0054	-0.0027	0.0064	0.0133	1.0000	0.7036	0.4602	0.0029
Prm15	-0.5936	0.0686	-0.0083	-0.0153	0.0028	-0.0036	0.0038	0.0008	0.0279	0.7036	1.0000	0.4426	-0.0093
Prm16	-0.3960	0.0658	-0.0175	-0.0021	0.0119	0.0033	0.0153	0.0009	0.0159	0.4602	0.4426	1.0000	-0.0206
Prm18	-0.0433	0.0042	0.0190	0.0023	-0.0068	0.0022	-0.0077	0.0013	0.0087	0.0029	-0.0093	-0.0206	1.0000

Robbery in circumstances of aggravation

Parameter Information

Parameter	Effect	gender	child_gosr	seifa5	ra3	birth_cohort	rob
Prm1	Intercept						
Prm2	gender	1					
Prm3	gender	0					
Prm4	child_gosr		1				
Prm5	child_gosr		0				
Prm6	seifa5			2			
Prm7	seifa5			3			
Prm8	seifa5			4			
Prm9	seifa5			5			
Prm10	seifa5			1			
Prm11	ra3				2		
Prm12	ra3				3		
Prm13	ra3				1		
Prm14	birth_cohort					2	
Prm15	birth_cohort					3	
Prm16	birth_cohort					4	
Prm17	birth_cohort					1	
Prm18	rob						1
Prm19	rob						0

Estimated Correlation Matrix

	Prm1	Prm2	Prm4	Prm6	Prm7	Prm8	Prm9	Prm11	Prm12	Prm14	Prm15	Prm16	Prm18
Prm1	1.0000	-0.4203	-0.2625	-0.3323	-0.3082	-0.3221	-0.3417	-0.3358	-0.2397	-0.6136	-0.5924	-0.3957	-0.0197
Prm2	-0.4203	1.0000	0.1016	0.0055	-0.0154	-0.0225	-0.0098	0.0156	0.0305	0.0369	0.0679	0.0670	-0.0459
Prm4	-0.2625	0.1016	1.0000	0.1113	0.1610	0.1767	0.1799	-0.0738	-0.4831	0.0030	-0.0110	-0.0197	-0.0042
Prm6	-0.3323	0.0055	0.1113	1.0000	0.3320	0.3101	0.2858	0.1147	0.0160	0.0049	-0.0139	-0.0006	-0.0016
Prm7	-0.3082	-0.0154	0.1610	0.3320	1.0000	0.2829	0.2666	0.0852	0.0374	0.0029	0.0026	0.0133	-0.0019
Prm8	-0.3221	-0.0225	0.1767	0.3101	0.2829	1.0000	0.2780	0.1670	0.0961	0.0057	-0.0035	0.0031	0.0014
Prm9	-0.3417	-0.0098	0.1799	0.2858	0.2666	0.2780	1.0000	0.2509	0.1669	-0.0034	0.0027	0.0137	-0.0130
Prm11	-0.3358	0.0156	-0.0738	0.1147	0.0852	0.1670	0.2509	1.0000	0.4145	0.0065	-0.0003	0.0000	0.0401
Prm12	-0.2397	0.0305	-0.4831	0.0160	0.0374	0.0961	0.1669	0.4145	1.0000	0.0138	0.0267	0.0137	0.0536
Prm14	-0.6136	0.0369	0.0030	0.0049	0.0029	0.0057	-0.0034	0.0065	0.0138	1.0000	0.7035	0.4604	-0.0142
Prm15	-0.5924	0.0679	-0.0110	-0.0139	0.0026	-0.0035	0.0027	-0.0003	0.0267	0.7035	1.0000	0.4434	-0.0419
Prm16	-0.3957	0.0670	-0.0197	-0.0006	0.0133	0.0031	0.0137	0.0000	0.0137	0.4604	0.4434	1.0000	-0.0411
Prm18	-0.0197	-0.0459	-0.0042	-0.0016	-0.0019	0.0014	-0.0130	0.0401	0.0536	-0.0142	-0.0419	-0.0411	1.0000

Dangerous driving causing death

Parameter Information

Parameter	Effect	gender	child_gosr	seifa5	ra3	birth_cohort	danger_death
Prm1	Intercept						
Prm2	gender	1					
Prm3	gender	0					
Prm4	child_gosr		1				
Prm5	child_gosr		0				
Prm6	seifa5			2			
Prm7	seifa5			3			
Prm8	seifa5			4			
Prm9	seifa5			5			
Prm10	seifa5			1			
Prm11	ra3				2		
Prm12	ra3				3		
Prm13	ra3				1		
Prm14	birth_cohort					2	
Prm15	birth_cohort					3	
Prm16	birth_cohort					4	
Prm17	birth_cohort					1	
Prm18	danger_death						1
Prm19	danger_death						0

Estimated Correlation Matrix

	Prm1	Prm2	Prm4	Prm6	Prm7	Prm8	Prm9	Prm11	Prm12	Prm14	Prm15	Prm16	Prm18
Prm1	1.0000	-0.4210	-0.2632	-0.3324	-0.3091	-0.3223	-0.3421	-0.3366	-0.2397	-0.6136	-0.5935	-0.3964	-0.0043
Prm2	-0.4210	1.0000	0.1015	0.0055	-0.0146	-0.0218	-0.0109	0.0189	0.0334	0.0356	0.0645	0.0631	-0.0126
Prm4	-0.2632	0.1015	1.0000	0.1118	0.1615	0.1773	0.1793	-0.0729	-0.4828	0.0031	-0.0112	-0.0205	0.0056
Prm6	-0.3324	0.0055	0.1118	1.0000	0.3321	0.3102	0.2860	0.1155	0.0162	0.0046	-0.0144	-0.0013	-0.0036
Prm7	-0.3091	-0.0146	0.1615	0.3321	1.0000	0.2831	0.2672	0.0864	0.0376	0.0034	0.0034	0.0142	-0.0252
Prm8	-0.3223	-0.0218	0.1773	0.3102	0.2831	1.0000	0.2781	0.1664	0.0962	0.0053	-0.0033	0.0033	-0.0019
Prm9	-0.3421	-0.0109	0.1793	0.2860	0.2672	0.2781	1.0000	0.2525	0.1687	-0.0037	0.0031	0.0137	-0.0169
Prm11	-0.3366	0.0189	-0.0729	0.1155	0.0864	0.1664	0.2525	1.0000	0.4132	0.0073	0.0018	0.0029	-0.0028
Prm12	-0.2397	0.0334	-0.4828	0.0162	0.0376	0.0962	0.1687	0.4132	1.0000	0.0146	0.0301	0.0168	-0.0027
Prm14	-0.6136	0.0356	0.0031	0.0046	0.0034	0.0053	-0.0037	0.0073	0.0146	1.0000	0.7036	0.4602	-0.0102

Estimated Correlation Matrix

	Prm1	Prm2	Prm4	Prm6	Prm7	Prm8	Prm9	Prm11	Prm12	Prm14	Prm15	Prm16	Prm18
Prm15	-0.5935	0.0645	-0.0112	-0.0144	0.0034	-0.0033	0.0031	0.0018	0.0301	0.7036	1.0000	0.4423	-0.0044
Prm16	-0.3964	0.0631	-0.0205	-0.0013	0.0142	0.0033	0.0137	0.0029	0.0168	0.4602	0.4423	1.0000	-0.0014
Prm18	-0.0043	-0.0126	0.0056	-0.0036	-0.0252	-0.0019	-0.0169	-0.0028	-0.0027	-0.0102	-0.0044	-0.0014	1.0000

Breach violence restraining order

Parameter Information

Parameter	Effect	gender	child_gosr	seifa5	ra3	birth_cohort	breach_violence
Prm1	Intercept						
Prm2	gender	1					
Prm3	gender	0					
Prm4	child_gosr		1				
Prm5	child_gosr		0				
Prm6	seifa5			2			
Prm7	seifa5			3			
Prm8	seifa5			4			
Prm9	seifa5			5			
Prm10	seifa5			1			
Prm11	ra3				2		
Prm12	ra3				3		
Prm13	ra3				1		
Prm14	birth_cohort					2	
Prm15	birth_cohort					3	
Prm16	birth_cohort					4	
Prm17	birth_cohort					1	
Prm18	breach_violence						1
Prm19	breach_violence						0

Estimated Correlation Matrix

	Prm1	Prm2	Prm4	Prm6	Prm7	Prm8	Prm9	Prm11	Prm12	Prm14	Prm15	Prm16	Prm18
Prm1	1.0000	-0.4225	-0.2590	-0.3314	-0.3081	-0.3233	-0.3429	-0.3372	-0.2406	-0.6124	-0.5937	-0.3973	-0.0647
Prm2	-0.4225	1.0000	0.1002	0.0052	-0.0145	-0.0216	-0.0090	0.0191	0.0351	0.0345	0.0653	0.0645	0.0373
Prm4	-0.2590	0.1002	1.0000	0.1128	0.1611	0.1760	0.1792	-0.0740	-0.4805	0.0012	-0.0149	-0.0226	-0.0431
Prm6	-0.3314	0.0052	0.1128	1.0000	0.3322	0.3106	0.2863	0.1149	0.0173	0.0030	-0.0152	-0.0015	-0.0037

Estimated Correlation Matrix													
	**Prm1**	**Prm2**	**Prm4**	**Prm6**	**Prm7**	**Prm8**	**Prm9**	**Prm11**	**Prm12**	**Prm14**	**Prm15**	**Prm16**	**Prm18**
Prm7	-0.3081	-0.0145	0.1611	0.3322	1.0000	0.2828	0.2665	0.0848	0.0365	0.0021	0.0031	0.0137	0.0116
Prm8	-0.3233	-0.0216	0.1760	0.3106	0.2828	1.0000	0.2785	0.1678	0.0974	0.0065	-0.0022	0.0038	0.0110
Prm9	-0.3429	-0.0090	0.1792	0.2863	0.2665	0.2785	1.0000	0.2527	0.1688	-0.0043	0.0029	0.0138	0.0168
Prm11	-0.3372	0.0191	-0.0740	0.1149	0.0848	0.1678	0.2527	1.0000	0.4138	0.0079	0.0035	0.0042	0.0166
Prm12	-0.2406	0.0351	-0.4805	0.0173	0.0365	0.0974	0.1688	0.4138	1.0000	0.0144	0.0301	0.0171	-0.0012
Prm14	-0.6124	0.0345	0.0012	0.0030	0.0021	0.0065	-0.0043	0.0079	0.0144	1.0000	0.7036	0.4604	0.0184
Prm15	-0.5937	0.0653	-0.0149	-0.0152	0.0031	-0.0022	0.0029	0.0035	0.0301	0.7036	1.0000	0.4429	0.0345
Prm16	-0.3973	0.0645	-0.0226	-0.0015	0.0137	0.0038	0.0138	0.0042	0.0171	0.4604	0.4429	1.0000	0.0274
Prm18	-0.0647	0.0373	-0.0431	-0.0037	0.0116	0.0110	0.0168	0.0166	-0.0012	0.0184	0.0345	0.0274	1.0000

Threats to harm, take control of conveyance or building

Parameter Information							
**Parameter**	**Effect**	**gender**	**child_gosr**	**seifa5**	**ra3**	**birth_cohort**	**threat_build**
Prm1	Intercept						
Prm2	gender	1					
Prm3	gender	0					
Prm4	child_gosr		1				
Prm5	child_gosr		0				
Prm6	seifa5			2			
Prm7	seifa5			3			
Prm8	seifa5			4			
Prm9	seifa5			5			
Prm10	seifa5			1			
Prm11	ra3				2		
Prm12	ra3				3		
Prm13	ra3				1		
Prm14	birth_cohort					2	
Prm15	birth_cohort					3	
Prm16	birth_cohort					4	
Prm17	birth_cohort					1	
Prm18	threat_build						1
Prm19	threat_build						0

Estimated Correlation Matrix

	Prm1	Prm2	Prm4	Prm6	Prm7	Prm8	Prm9	Prm11	Prm12	Prm14	Prm15	Prm16	Prm18
Prm1	1.0000	-0.4216	-0.2631	-0.3329	-0.3084	-0.3227	-0.3432	-0.3354	-0.2407	-0.6128	-0.5925	-0.3968	-0.0522
Prm2	-0.4216	1.0000	0.1027	0.0062	-0.0151	-0.0220	-0.0104	0.0185	0.0347	0.0355	0.0643	0.0639	0.0076
Prm4	-0.2631	0.1027	1.0000	0.1129	0.1618	0.1785	0.1804	-0.0738	-0.4829	0.0026	-0.0120	-0.0211	0.0013
Prm6	-0.3329	0.0062	0.1129	1.0000	0.3323	0.3106	0.2864	0.1147	0.0161	0.0043	-0.0145	-0.0007	0.0150
Prm7	-0.3084	-0.0151	0.1618	0.3323	1.0000	0.2832	0.2669	0.0844	0.0374	0.0026	0.0026	0.0137	0.0100
Prm8	-0.3227	-0.0220	0.1785	0.3106	0.2832	1.0000	0.2786	0.1660	0.0956	0.0054	-0.0034	0.0033	0.0154
Prm9	-0.3432	-0.0104	0.1804	0.2864	0.2669	0.2786	1.0000	0.2515	0.1681	-0.0031	0.0029	0.0144	0.0237
Prm11	-0.3354	0.0185	-0.0738	0.1147	0.0844	0.1660	0.2515	1.0000	0.4134	0.0077	0.0022	0.0033	-0.0009
Prm12	-0.2407	0.0347	-0.4829	0.0161	0.0374	0.0956	0.1681	0.4134	1.0000	0.0154	0.0307	0.0186	0.0139
Prm14	-0.6128	0.0355	0.0026	0.0043	0.0026	0.0054	-0.0031	0.0077	0.0154	1.0000	0.7036	0.4602	0.0014
Prm15	-0.5925	0.0643	-0.0120	-0.0145	0.0026	-0.0034	0.0029	0.0022	0.0307	0.7036	1.0000	0.4423	0.0030
Prm16	-0.3968	0.0639	-0.0211	-0.0007	0.0137	0.0033	0.0144	0.0033	0.0186	0.4602	0.4423	1.0000	0.0087
Prm18	-0.0522	0.0076	0.0013	0.0150	0.0100	0.0154	0.0237	-0.0009	0.0139	0.0014	0.0030	0.0087	1.0000

Burglary and commit

Parameter Information

Parameter	Effect	gender	child_gosr	seifa5	ra3	birth_cohort	burg_commit
Prm1	Intercept						
Prm2	gender	1					
Prm3	gender	0					
Prm4	child_gosr		1				
Prm5	child_gosr		0				
Prm6	seifa5			2			
Prm7	seifa5			3			
Prm8	seifa5			4			
Prm9	seifa5			5			
Prm10	seifa5			1			
Prm11	ra3				2		
Prm12	ra3				3		
Prm13	ra3				1		
Prm14	birth_cohort					2	
Prm15	birth_cohort					3	
Prm16	birth_cohort					4	
Prm17	birth_cohort					1	

Parameter Information							
Parameter	Effect	gender	child_gosr	seifa5	ra3	birth_cohort	burg_commit
Prm18	burg_commit						1
Prm19	burg_commit						0

Estimated Correlation Matrix													
	Prm1	Prm2	Prm4	Prm6	Prm7	Prm8	Prm9	Prm11	Prm12	Prm14	Prm15	Prm16	Prm18
Prm1	1.0000	-0.4223	-0.2643	-0.3313	-0.3081	-0.3223	-0.3422	-0.3356	-0.2383	-0.6138	-0.5940	-0.3976	-0.0375
Prm2	-0.4223	1.0000	0.1035	0.0059	-0.0150	-0.0226	-0.0104	0.0191	0.0335	0.0368	0.0663	0.0646	-0.0041
Prm4	-0.2643	0.1035	1.0000	0.1119	0.1619	0.1768	0.1798	-0.0734	-0.4838	0.0041	-0.0102	-0.0191	0.0056
Prm6	-0.3313	0.0059	0.1119	1.0000	0.3320	0.3100	0.2857	0.1146	0.0155	0.0042	-0.0148	-0.0012	-0.0059
Prm7	-0.3081	-0.0150	0.1619	0.3320	1.0000	0.2829	0.2667	0.0852	0.0369	0.0031	0.0026	0.0136	-0.0035
Prm8	-0.3223	-0.0226	0.1768	0.3100	0.2829	1.0000	0.2781	0.1667	0.0958	0.0061	-0.0028	0.0039	0.0112
Prm9	-0.3422	-0.0104	0.1798	0.2857	0.2667	0.2781	1.0000	0.2523	0.1679	-0.0032	0.0031	0.0143	0.0039
Prm11	-0.3356	0.0191	-0.0734	0.1146	0.0852	0.1667	0.2523	1.0000	0.4133	0.0076	0.0019	0.0027	-0.0092
Prm12	-0.2383	0.0335	-0.4838	0.0155	0.0369	0.0958	0.1679	0.4133	1.0000	0.0143	0.0294	0.0175	-0.0079
Prm14	-0.6138	0.0368	0.0041	0.0042	0.0031	0.0061	-0.0032	0.0076	0.0143	1.0000	0.7036	0.4603	0.0056
Prm15	-0.5940	0.0663	-0.0102	-0.0148	0.0026	-0.0028	0.0031	0.0019	0.0294	0.7036	1.0000	0.4426	0.0145
Prm16	-0.3976	0.0646	-0.0191	-0.0012	0.0136	0.0039	0.0143	0.0027	0.0175	0.4603	0.4426	1.0000	0.0164
Prm18	-0.0375	-0.0041	0.0056	-0.0059	-0.0035	0.0112	0.0039	-0.0092	-0.0079	0.0056	0.0145	0.0164	1.0000

Breach police restraining order

Parameter Information							
Parameter	Effect	gender	child_gosr	seifa5	ra3	birth_cohort	breach_police
Prm1	Intercept						
Prm2	gender	1					
Prm3	gender	0					
Prm4	child_gosr		1				
Prm5	child_gosr		0				
Prm6	seifa5			2			
Prm7	seifa5			3			
Prm8	seifa5			4			
Prm9	seifa5			5			
Prm10	seifa5			1			
Prm11	ra3				2		

Parameter Information

Parameter	Effect	gender	child_gosr	seifa5	ra3	birth_cohort	breach_police
Prm12	ra3				3		
Prm13	ra3				1		
Prm14	birth_cohort					2	
Prm15	birth_cohort					3	
Prm16	birth_cohort					4	
Prm17	birth_cohort					1	
Prm18	breach_police						1
Prm19	breach_police						0

Estimated Correlation Matrix

	Prm1	Prm2	Prm4	Prm6	Prm7	Prm8	Prm9	Prm11	Prm12	Prm14	Prm15	Prm16	Prm18
Prm1	1.0000	-0.4221	-0.2627	-0.3332	-0.3091	-0.3223	-0.3425	-0.3355	-0.2415	-0.6129	-0.5917	-0.3965	-0.0315
Prm2	-0.4221	1.0000	0.1009	0.0069	-0.0129	-0.0211	-0.0098	0.0175	0.0353	0.0346	0.0626	0.0631	0.0337
Prm4	-0.2627	0.1009	1.0000	0.1118	0.1616	0.1771	0.1796	-0.0720	-0.4804	0.0039	-0.0097	-0.0203	-0.0372
Prm6	-0.3332	0.0069	0.1118	1.0000	0.3322	0.3103	0.2861	0.1159	0.0162	0.0051	-0.0141	-0.0009	0.0047
Prm7	-0.3091	-0.0129	0.1616	0.3322	1.0000	0.2830	0.2668	0.0862	0.0368	0.0026	0.0011	0.0138	0.0140
Prm8	-0.3223	-0.0211	0.1771	0.3103	0.2830	1.0000	0.2781	0.1671	0.0960	0.0055	-0.0042	0.0032	0.0052
Prm9	-0.3425	-0.0098	0.1796	0.2861	0.2668	0.2781	1.0000	0.2527	0.1687	-0.0034	0.0017	0.0134	0.0082
Prm11	-0.3355	0.0175	-0.0720	0.1159	0.0862	0.1671	0.2527	1.0000	0.4132	0.0072	0.0012	0.0025	-0.0084
Prm12	-0.2415	0.0353	-0.4804	0.0162	0.0368	0.0960	0.1687	0.4132	1.0000	0.0158	0.0315	0.0188	-0.0006
Prm14	-0.6129	0.0346	0.0039	0.0051	0.0026	0.0055	-0.0034	0.0072	0.0158	1.0000	0.7036	0.4602	-0.0109
Prm15	-0.5917	0.0626	-0.0097	-0.0141	0.0011	-0.0042	0.0017	0.0012	0.0315	0.7036	1.0000	0.4421	-0.0226
Prm16	-0.3965	0.0631	-0.0203	-0.0009	0.0138	0.0032	0.0134	0.0025	0.0188	0.4602	0.4421	1.0000	0.0009
Prm18	-0.0315	0.0337	-0.0372	0.0047	0.0140	0.0052	0.0082	-0.0084	-0.0006	-0.0109	-0.0226	0.0009	1.0000

Robbery

Parameter Information

Parameter	Effect	gender	child_gosr	seifa5	ra3	birth_cohort	rob_agg
Prm1	Intercept						
Prm2	gender	1					
Prm3	gender	0					
Prm4	child_gosr		1				
Prm5	child_gosr		0				

Parameter Information

Parameter	Effect	gender	child_gosr	seifa5	ra3	birth_cohort	rob_agg
Prm6	seifa5			2			
Prm7	seifa5			3			
Prm8	seifa5			4			
Prm9	seifa5			5			
Prm10	seifa5			1			
Prm11	ra3				2		
Prm12	ra3				3		
Prm13	ra3				1		
Prm14	birth_cohort					2	
Prm15	birth_cohort					3	
Prm16	birth_cohort					4	
Prm17	birth_cohort					1	
Prm18	rob_agg						1
Prm19	rob_agg						0

Estimated Correlation Matrix

	Prm1	Prm2	Prm4	Prm6	Prm7	Prm8	Prm9	Prm11	Prm12	Prm14	Prm15	Prm16	Prm18
Prm1	1.0000	-0.4212	-0.2629	-0.3322	-0.3083	-0.3222	-0.3420	-0.3356	-0.2398	-0.6137	-0.5930	-0.3965	-0.0082
Prm2	-0.4212	1.0000	0.1018	0.0060	-0.0149	-0.0221	-0.0094	0.0170	0.0326	0.0365	0.0661	0.0648	-0.0303
Prm4	-0.2629	0.1018	1.0000	0.1116	0.1613	0.1767	0.1801	-0.0734	-0.4825	0.0030	-0.0112	-0.0200	-0.0049
Prm6	-0.3322	0.0060	0.1116	1.0000	0.3320	0.3101	0.2858	0.1143	0.0155	0.0050	-0.0137	-0.0009	-0.0103
Prm7	-0.3083	-0.0149	0.1613	0.3320	1.0000	0.2828	0.2665	0.0851	0.0369	0.0029	0.0027	0.0136	-0.0052
Prm8	-0.3222	-0.0221	0.1767	0.3101	0.2828	1.0000	0.2780	0.1668	0.0963	0.0057	-0.0033	0.0032	-0.0015
Prm9	-0.3420	-0.0094	0.1801	0.2858	0.2665	0.2780	1.0000	0.2511	0.1672	-0.0034	0.0032	0.0136	-0.0206
Prm11	-0.3356	0.0170	-0.0734	0.1143	0.0851	0.1668	0.2511	1.0000	0.4138	0.0066	0.0005	0.0020	0.0261
Prm12	-0.2398	0.0326	-0.4825	0.0155	0.0369	0.0963	0.1672	0.4138	1.0000	0.0142	0.0284	0.0160	0.0360
Prm14	-0.6137	0.0365	0.0030	0.0050	0.0029	0.0057	-0.0034	0.0066	0.0142	1.0000	0.7036	0.4605	-0.0153
Prm15	-0.5930	0.0661	-0.0112	-0.0137	0.0027	-0.0033	0.0032	0.0005	0.0284	0.7036	1.0000	0.4428	-0.0322
Prm16	-0.3965	0.0648	-0.0200	-0.0009	0.0136	0.0032	0.0136	0.0020	0.0160	0.4605	0.4428	1.0000	-0.0234
Prm18	-0.0082	-0.0303	-0.0049	-0.0103	-0.0052	-0.0015	-0.0206	0.0261	0.0360	-0.0153	-0.0322	-0.0234	1.0000

Assault serious

Parameter Information

Parameter	Effect	gender	child_gosr	seifa5	ra3	birth_cohort	assault_serious
Prm1	Intercept						
Prm2	gender	1					
Prm3	gender	0					
Prm4	child_gosr		1				
Prm5	child_gosr		0				
Prm6	seifa5			2			
Prm7	seifa5			3			
Prm8	seifa5			4			
Prm9	seifa5			5			
Prm10	seifa5			1			
Prm11	ra3				2		
Prm12	ra3				3		
Prm13	ra3				1		
Prm14	birth_cohort					2	
Prm15	birth_cohort					3	
Prm16	birth_cohort					4	
Prm17	birth_cohort					1	
Prm18	assault_serious						1
Prm19	assault_serious						0

Estimated Correlation Matrix

	Prm1	Prm2	Prm4	Prm6	Prm7	Prm8	Prm9	Prm11	Prm12	Prm14	Prm15	Prm16	Prm18
Prm1	1.0000	-0.4190	-0.2613	-0.3310	-0.3073	-0.3212	-0.3417	-0.3371	-0.2428	-0.6159	-0.5963	-0.3996	-0.0956
Prm2	-0.4190	1.0000	0.1019	0.0060	-0.0152	-0.0223	-0.0103	0.0181	0.0338	0.0352	0.0640	0.0632	-0.0067
Prm4	-0.2613	0.1019	1.0000	0.1119	0.1615	0.1771	0.1800	-0.0732	-0.4820	0.0024	-0.0123	-0.0207	-0.0050
Prm6	-0.3310	0.0060	0.1119	1.0000	0.3320	0.3102	0.2858	0.1146	0.0161	0.0049	-0.0140	-0.0009	-0.0009
Prm7	-0.3073	-0.0152	0.1615	0.3320	1.0000	0.2830	0.2667	0.0854	0.0373	0.0029	0.0027	0.0137	0.0034
Prm8	-0.3212	-0.0223	0.1771	0.3102	0.2830	1.0000	0.2782	0.1669	0.0965	0.0059	-0.0031	0.0035	0.0028
Prm9	-0.3417	-0.0103	0.1800	0.2858	0.2667	0.2782	1.0000	0.2522	0.1686	-0.0029	0.0031	0.0141	0.0088
Prm11	-0.3371	0.0181	-0.0732	0.1146	0.0854	0.1669	0.2522	1.0000	0.4140	0.0092	0.0040	0.0044	0.0291
Prm12	-0.2428	0.0338	-0.4820	0.0161	0.0373	0.0965	0.1686	0.4140	1.0000	0.0175	0.0331	0.0195	0.0409
Prm14	-0.6159	0.0352	0.0024	0.0049	0.0029	0.0059	-0.0029	0.0092	0.0175	1.0000	0.7050	0.4622	0.0653
Prm15	-0.5963	0.0640	-0.0123	-0.0140	0.0027	-0.0031	0.0031	0.0040	0.0331	0.7050	1.0000	0.4446	0.0786
Prm16	-0.3996	0.0632	-0.0207	-0.0009	0.0137	0.0035	0.0141	0.0044	0.0195	0.4622	0.4446	1.0000	0.0552
Prm18	-0.0956	-0.0067	-0.0050	-0.0009	0.0034	0.0028	0.0088	0.0291	0.0409	0.0653	0.0786	0.0552	1.0000

Going armed in public to cause fear

Parameter Information

Parameter	Effect	gender	child_gosr	seifa5	ra3	birth_cohort	going
Prm1	Intercept						
Prm2	gender	1					
Prm3	gender	0					
Prm4	child_gosr		1				
Prm5	child_gosr		0				
Prm6	seifa5			2			
Prm7	seifa5			3			
Prm8	seifa5			4			
Prm9	seifa5			5			
Prm10	seifa5			1			
Prm11	ra3				2		
Prm12	ra3				3		
Prm13	ra3				1		
Prm14	birth_cohort					2	
Prm15	birth_cohort					3	
Prm16	birth_cohort					4	
Prm17	birth_cohort					1	
Prm18	going						1
Prm19	going						0

Estimated Correlation Matrix

	Prm1	Prm2	Prm4	Prm6	Prm7	Prm8	Prm9	Prm11	Prm12	Prm14	Prm15	Prm16	Prm18
Prm1	1.0000	-0.4213	-0.2630	-0.3325	-0.3083	-0.3221	-0.3421	-0.3361	-0.2400	-0.6138	-0.5932	-0.3970	-0.0045
Prm2	-0.4213	1.0000	0.1019	0.0057	-0.0153	-0.0226	-0.0107	0.0180	0.0332	0.0366	0.0660	0.0650	-0.0323
Prm4	-0.2630	0.1019	1.0000	0.1116	0.1615	0.1768	0.1798	-0.0731	-0.4824	0.0032	-0.0114	-0.0202	-0.0043
Prm6	-0.3325	0.0057	0.1116	1.0000	0.3320	0.3101	0.2858	0.1146	0.0161	0.0051	-0.0140	-0.0002	-0.0009
Prm7	-0.3083	-0.0153	0.1615	0.3320	1.0000	0.2829	0.2666	0.0852	0.0370	0.0027	0.0029	0.0138	-0.0017
Prm8	-0.3221	-0.0226	0.1768	0.3101	0.2829	1.0000	0.2781	0.1672	0.0966	0.0055	-0.0034	0.0033	0.0026
Prm9	-0.3421	-0.0107	0.1798	0.2858	0.2666	0.2781	1.0000	0.2521	0.1684	-0.0036	0.0019	0.0139	0.0047
Prm11	-0.3361	0.0180	-0.0731	0.1146	0.0852	0.1672	0.2521	1.0000	0.4134	0.0072	0.0016	0.0029	0.0086
Prm12	-0.2400	0.0332	-0.4824	0.0161	0.0370	0.0966	0.1684	0.4134	1.0000	0.0145	0.0292	0.0172	0.0215
Prm14	-0.6138	0.0366	0.0032	0.0051	0.0027	0.0055	-0.0036	0.0072	0.0145	1.0000	0.7037	0.4604	-0.0147

Estimated Correlation Matrix

	Prm1	Prm2	Prm4	Prm6	Prm7	Prm8	Prm9	Prm11	Prm12	Prm14	Prm15	Prm16	Prm18
Prm15	-0.5932	0.0660	-0.0114	-0.0140	0.0029	-0.0034	0.0019	0.0016	0.0292	0.7037	1.0000	0.4427	-0.0276
Prm16	-0.3970	0.0650	-0.0202	-0.0002	0.0138	0.0033	0.0139	0.0029	0.0172	0.4604	0.4427	1.0000	-0.0234
Prm18	-0.0045	-0.0323	-0.0043	-0.0009	-0.0017	0.0026	0.0047	0.0086	0.0215	-0.0147	-0.0276	-0.0234	1.0000

Act or omission with intent to harm and causing bodily harm

Parameter Information

Parameter	Effect	gender	child_gosr	seifa5	ra3	birth_cohort	act_cause
Prm1	Intercept						
Prm2	gender	1					
Prm3	gender	0					
Prm4	child_gosr		1				
Prm5	child_gosr		0				
Prm6	seifa5			2			
Prm7	seifa5			3			
Prm8	seifa5			4			
Prm9	seifa5			5			
Prm10	seifa5			1			
Prm11	ra3				2		
Prm12	ra3				3		
Prm13	ra3				1		
Prm14	birth_cohort					2	
Prm15	birth_cohort					3	
Prm16	birth_cohort					4	
Prm17	birth_cohort					1	
Prm18	act_cause						1
Prm19	act_cause						0

Estimated Correlation Matrix

	Prm1	Prm2	Prm4	Prm6	Prm7	Prm8	Prm9	Prm11	Prm12	Prm14	Prm15	Prm16	Prm18
Prm1	1.0000	-0.4215	-0.2631	-0.3327	-0.3085	-0.3223	-0.3427	-0.3361	-0.2407	-0.6135	-0.5929	-0.3963	-0.0120
Prm2	-0.4215	1.0000	0.1021	0.0059	-0.0149	-0.0221	-0.0101	0.0176	0.0339	0.0360	0.0653	0.0639	-0.0179
Prm4	-0.2631	0.1021	1.0000	0.1119	0.1616	0.1773	0.1800	-0.0736	-0.4821	0.0030	-0.0112	-0.0203	-0.0059
Prm6	-0.3327	0.0059	0.1119	1.0000	0.3321	0.3102	0.2860	0.1149	0.0163	0.0045	-0.0142	-0.0016	0.0129

Estimated Correlation Matrix													
	**Prm1**	**Prm2**	**Prm4**	**Prm6**	**Prm7**	**Prm8**	**Prm9**	**Prm11**	**Prm12**	**Prm14**	**Prm15**	**Prm16**	**Prm18**
Prm7	-0.3085	-0.0149	0.1616	0.3321	1.0000	0.2830	0.2667	0.0856	0.0374	0.0025	0.0023	0.0136	0.0072
Prm8	-0.3223	-0.0221	0.1773	0.3102	0.2830	1.0000	0.2781	0.1669	0.0961	0.0055	-0.0035	0.0031	0.0060
Prm9	-0.3427	-0.0101	0.1800	0.2860	0.2667	0.2781	1.0000	0.2523	0.1688	-0.0038	0.0022	0.0133	0.0196
Prm11	-0.3361	0.0176	-0.0736	0.1149	0.0856	0.1669	0.2523	1.0000	0.4140	0.0069	0.0015	0.0022	0.0288
Prm12	-0.2407	0.0339	-0.4821	0.0163	0.0374	0.0961	0.1688	0.4140	1.0000	0.0147	0.0293	0.0172	0.0322
Prm14	-0.6135	0.0360	0.0030	0.0045	0.0025	0.0055	-0.0038	0.0069	0.0147	1.0000	0.7035	0.4604	-0.0112
Prm15	-0.5929	0.0653	-0.0112	-0.0142	0.0023	-0.0035	0.0022	0.0015	0.0293	0.7035	1.0000	0.4428	-0.0333
Prm16	-0.3963	0.0639	-0.0203	-0.0016	0.0136	0.0031	0.0133	0.0022	0.0172	0.4604	0.4428	1.0000	-0.0267
Prm18	-0.0120	-0.0179	-0.0059	0.0129	0.0072	0.0060	0.0196	0.0288	0.0322	-0.0112	-0.0333	-0.0267	1.0000

Hospital admission relative risk models (Figure 1):

Assault causing bodily harm

Parameter Information							
**Parameter**	**Effect**	**gender**	**child_gosr**	**seifa5**	**ra3**	**birth_cohort**	**assault_harm**
Prm1	Intercept						
Prm2	gender	1					
Prm3	gender	0					
Prm4	child_gosr		1				
Prm5	child_gosr		0				
Prm6	seifa5			2			
Prm7	seifa5			3			
Prm8	seifa5			4			
Prm9	seifa5			5			
Prm10	seifa5			1			
Prm11	ra3				2		
Prm12	ra3				3		
Prm13	ra3				1		
Prm14	birth_cohort					2	
Prm15	birth_cohort					3	
Prm16	birth_cohort					4	
Prm17	birth_cohort					1	
Prm18	assault_harm						1

Parameter Information							
Parameter	Effect	gender	child_gosr	seifa5	ra3	birth_cohort	assault_harm
Prm19	assault_harm						0

Estimated Correlation Matrix													
	Prm1	Prm2	Prm4	Prm6	Prm7	Prm8	Prm9	Prm11	Prm12	Prm14	Prm15	Prm16	Prm18
Prm1	1.0000	-0.4073	-0.2557	-0.3330	-0.3007	-0.3202	-0.3455	-0.2805	-0.1989	-0.6426	-0.6228	-0.4583	-0.1484
Prm2	-0.4073	1.0000	0.1135	0.0097	-0.0114	-0.0155	-0.0098	0.0144	0.0370	0.0358	0.0596	0.0650	-0.0987
Prm4	-0.2557	0.1135	1.0000	0.1156	0.1640	0.1877	0.2013	-0.0729	-0.4907	0.0069	-0.0067	-0.0202	-0.0339
Prm6	-0.3330	0.0097	0.1156	1.0000	0.3636	0.3526	0.3366	0.1120	0.0180	0.0066	-0.0119	0.0007	-0.0197
Prm7	-0.3007	-0.0114	0.1640	0.3636	1.0000	0.3138	0.3062	0.0792	0.0363	0.0029	0.0038	0.0154	-0.0167
Prm8	-0.3202	-0.0155	0.1877	0.3526	0.3138	1.0000	0.3265	0.1626	0.0939	0.0057	-0.0017	0.0029	-0.0240
Prm9	-0.3455	-0.0098	0.2013	0.3366	0.3062	0.3265	1.0000	0.2450	0.1632	-0.0009	0.0034	0.0158	-0.0146
Prm11	-0.2805	0.0144	-0.0729	0.1120	0.0792	0.1626	0.2450	1.0000	0.3714	0.0063	0.0002	0.0061	0.0191
Prm12	-0.1989	0.0370	-0.4907	0.0180	0.0363	0.0939	0.1632	0.3714	1.0000	0.0113	0.0223	0.0193	0.0009
Prm14	-0.6426	0.0358	0.0069	0.0066	0.0029	0.0057	-0.0009	0.0063	0.0113	1.0000	0.7521	0.5386	-0.0230
Prm15	-0.6228	0.0596	-0.0067	-0.0119	0.0038	-0.0017	0.0034	0.0002	0.0223	0.7521	1.0000	0.5177	-0.0051
Prm16	-0.4583	0.0650	-0.0202	0.0007	0.0154	0.0029	0.0158	0.0061	0.0193	0.5386	0.5177	1.0000	0.0034
Prm18	-0.1484	-0.0987	-0.0339	-0.0197	-0.0167	-0.0240	-0.0146	0.0191	0.0009	-0.0230	-0.0051	0.0034	1.0000

Common assault

Parameter Information							
Parameter	Effect	gender	child_gosr	seifa5	ra3	birth_cohort	common_assault
Prm1	Intercept						
Prm2	gender	1					
Prm3	gender	0					
Prm4	child_gosr		1				
Prm5	child_gosr		0				
Prm6	seifa5			2			
Prm7	seifa5			3			
Prm8	seifa5			4			
Prm9	seifa5			5			
Prm10	seifa5			1			
Prm11	ra3				2		
Prm12	ra3				3		

Parameter	Effect	gender	child_gosr	seifa5	ra3	birth_cohort	common_assault
Prm13	ra3				1		
Prm14	birth_cohort					2	
Prm15	birth_cohort					3	
Prm16	birth_cohort					4	
Prm17	birth_cohort					1	
Prm18	common_assault						1
Prm19	common_assault						0

Estimated Correlation Matrix

	Prm1	Prm2	Prm4	Prm6	Prm7	Prm8	Prm9	Prm11	Prm12	Prm14	Prm15	Prm16	Prm18
Prm1	1.0000	-0.4301	-0.2577	-0.3392	-0.3098	-0.3303	-0.3547	-0.2816	-0.2036	-0.6439	-0.6214	-0.4526	-0.1381
Prm2	-0.4301	1.0000	0.1030	0.0131	-0.0095	-0.0152	-0.0044	0.0274	0.0430	0.0293	0.0513	0.0567	0.0589
Prm4	-0.2577	0.1030	1.0000	0.1144	0.1640	0.1910	0.2021	-0.0768	-0.4835	0.0042	-0.0108	-0.0180	-0.0003
Prm6	-0.3392	0.0131	0.1144	1.0000	0.3634	0.3521	0.3359	0.1095	0.0203	0.0047	-0.0101	-0.0036	0.0093
Prm7	-0.3098	-0.0095	0.1640	0.3634	1.0000	0.3151	0.3076	0.0817	0.0416	0.0039	0.0077	0.0195	0.0076
Prm8	-0.3303	-0.0152	0.1910	0.3521	0.3151	1.0000	0.3282	0.1632	0.0972	0.0068	0.0008	0.0063	0.0086
Prm9	-0.3547	-0.0044	0.2021	0.3359	0.3076	0.3282	1.0000	0.2456	0.1686	-0.0013	0.0075	0.0141	0.0088
Prm11	-0.2816	0.0274	-0.0768	0.1095	0.0817	0.1632	0.2456	1.0000	0.3768	0.0078	0.0030	0.0001	-0.0093
Prm12	-0.2036	0.0430	-0.4835	0.0203	0.0416	0.0972	0.1686	0.3768	1.0000	0.0068	0.0204	0.0096	0.0058
Prm14	-0.6439	0.0293	0.0042	0.0047	0.0039	0.0068	-0.0013	0.0078	0.0068	1.0000	0.7521	0.5387	-0.0070
Prm15	-0.6214	0.0513	-0.0108	-0.0101	0.0077	0.0008	0.0075	0.0030	0.0204	0.7521	1.0000	0.5175	-0.0085
Prm16	-0.4526	0.0567	-0.0180	-0.0036	0.0195	0.0063	0.0141	0.0001	0.0096	0.5387	0.5175	1.0000	-0.0035
Prm18	-0.1381	0.0589	-0.0003	0.0093	0.0076	0.0086	0.0088	-0.0093	0.0058	-0.0070	-0.0085	-0.0035	1.0000

Wounding

Parameter Information

Parameter	Effect	gender	child_gosr	seifa5	ra3	birth_cohort	wound
Prm1	Intercept						
Prm2	gender	1					
Prm3	gender	0					
Prm4	child_gosr		1				
Prm5	child_gosr		0				
Prm6	seifa5			2			

Parameter Information							
Parameter	Effect	gender	child_gos	rseifa5	ra3	birth_cohort	wound
Prm7	seifa5			3			
Prm8	seifa5			4			
Prm9	seifa5			5			
Prm10	seifa5			1			
Prm11	ra3				2		
Prm12	ra3				3		
Prm13	ra3				1		
Prm14	birth_cohort					2	
Prm15	birth_cohort					3	
Prm16	birth_cohort					4	
Prm17	birth_cohort					1	
Prm18	wound						1
Prm19	wound						0

Estimated Correlation Matrix													
	**Prm1**	**Prm2**	**Prm4**	**Prm6**	**Prm7**	**Prm8**	**Prm9**	**Prm11**	**Prm12**	**Prm14**	**Prm15**	**Prm16**	**Prm18**
Prm1	1.0000	-0.4241	-0.2493	-0.3402	-0.3065	-0.3320	-0.3516	-0.2827	-0.2039	-0.6539	-0.6293	-0.4612	-0.0677
Prm2	-0.4241	1.0000	0.1101	0.0129	-0.0098	-0.0155	0.0001	0.0212	0.0414	0.0329	0.0597	0.0639	-0.0604
Prm4	-0.2493	0.1101	1.0000	0.1137	0.1604	0.1875	0.1985	-0.0798	-0.4932	0.0030	-0.0103	-0.0218	-0.0840
Prm6	-0.3402	0.0129	0.1137	1.0000	0.3628	0.3518	0.3360	0.1133	0.0180	0.0092	-0.0092	-0.0002	-0.0141
Prm7	-0.3065	-0.0098	0.1604	0.3628	1.0000	0.3136	0.3058	0.0789	0.0393	0.0036	0.0054	0.0203	-0.0098
Prm8	-0.3320	-0.0155	0.1875	0.3518	0.3136	1.0000	0.3262	0.1595	0.0948	0.0146	0.0039	0.0083	-0.0076
Prm9	-0.3516	0.0001	0.1985	0.3360	0.3058	0.3262	1.0000	0.2455	0.1657	-0.0029	0.0013	0.0180	-0.0170
Prm11	-0.2827	0.0212	-0.0798	0.1133	0.0789	0.1595	0.2455	1.0000	0.3738	0.0069	0.0027	0.0034	0.0187
Prm12	-0.2039	0.0414	-0.4932	0.0180	0.0393	0.0948	0.1657	0.3738	1.0000	0.0157	0.0300	0.0211	-0.0219
Prm14	-0.6539	0.0329	0.0030	0.0092	0.0036	0.0146	-0.0029	0.0069	0.0157	1.0000	0.7520	0.5387	0.0152
Prm15	-0.6293	0.0597	-0.0103	-0.0092	0.0054	0.0039	0.0013	0.0027	0.0300	0.7520	1.0000	0.5177	-0.0091
Prm16	-0.4612	0.0639	-0.0218	-0.0002	0.0203	0.0083	0.0180	0.0034	0.0211	0.5387	0.5177	1.0000	0.0010
Prm18	-0.0677	-0.0604	-0.0840	-0.0141	-0.0098	-0.0076	-0.0170	0.0187	-0.0219	0.0152	-0.0091	0.0010	1.0000

Grievous bodily harm

Parameter Information

Parameter	Effect	gender	child_gosr	seifa5	ra3	birth_cohort	grievous
Prm1	Intercept						
Prm2	gender	1					
Prm3	gender	0					
Prm4	child_gosr		1				
Prm5	child_gosr		0				
Prm6	seifa5			2			
Prm7	seifa5			3			
Prm8	seifa5			4			
Prm9	seifa5			5			
Prm10	seifa5			1			
Prm11	ra3				2		
Prm12	ra3				3		
Prm13	ra3				1		
Prm14	birth_cohort					2	
Prm15	birth_cohort					3	
Prm16	birth_cohort					4	
Prm17	birth_cohort					1	
Prm18	grievous						1
Prm19	grievous						0

Estimated Correlation Matrix

	Prm1	Prm2	Prm4	Prm6	Prm7	Prm8	Prm9	Prm11	Prm12	Prm14	Prm15	Prm16	Prm18
Prm1	1.0000	-0.4157	-0.2605	-0.3379	-0.3071	-0.3236	-0.3505	-0.2842	-0.2036	-0.6520	-0.6288	-0.4600	-0.0080
Prm2	-0.4157	1.0000	0.1099	0.0106	-0.0154	-0.0158	-0.0100	0.0121	0.0333	0.0344	0.0580	0.0624	-0.1817
Prm4	-0.2605	0.1099	1.0000	0.1170	0.1653	0.1833	0.1989	-0.0773	-0.4974	0.0017	-0.0150	-0.0228	0.0130
Prm6	-0.3379	0.0106	0.1170	1.0000	0.3631	0.3529	0.3370	0.1081	0.0145	0.0029	-0.0128	0.0040	-0.0186
Prm7	-0.3071	-0.0154	0.1653	0.3631	1.0000	0.3118	0.3044	0.0707	0.0307	0.0071	0.0066	0.0177	0.0031
Prm8	-0.3236	-0.0158	0.1833	0.3529	0.3118	1.0000	0.3268	0.1650	0.0865	0.0042	-0.0007	0.0061	-0.0323
Prm9	-0.3505	-0.0100	0.1989	0.3370	0.3044	0.3268	1.0000	0.2440	0.1616	0.0023	0.0054	0.0229	-0.0416
Prm11	-0.2842	0.0121	-0.0773	0.1081	0.0707	0.1650	0.2440	1.0000	0.3744	0.0086	0.0082	0.0048	0.0319
Prm12	-0.2036	0.0333	-0.4974	0.0145	0.0307	0.0865	0.1616	0.3744	1.0000	0.0134	0.0347	0.0171	0.0218
Prm14	-0.6520	0.0344	0.0017	0.0029	0.0071	0.0042	0.0023	0.0086	0.0134	1.0000	0.7526	0.5394	-0.0343
Prm15	-0.6288	0.0580	-0.0150	-0.0128	0.0066	-0.0007	0.0054	0.0082	0.0347	0.7526	1.0000	0.5180	-0.0552
Prm16	-0.4600	0.0624	-0.0228	0.0040	0.0177	0.0061	0.0229	0.0048	0.0171	0.5394	0.5180	1.0000	-0.0396
Prm18	-0.0080	-0.1817	0.0130	-0.0186	0.0031	-0.0323	-0.0416	0.0319	0.0218	-0.0343	-0.0552	-0.0396	1.0000

Dangerous driving causing grievous bodily harm

Parameter Information

Parameter	Effect	gender	child_gosr	seifa5	ra3	birth_cohort	danger_harm
Prm1	Intercept						
Prm2	gender	1					
Prm3	gender	0					
Prm4	child_gosr		1				
Prm5	child_gosr		0				
Prm6	seifa5			2			
Prm7	seifa5			3			
Prm8	seifa5			4			
Prm9	seifa5			5			
Prm10	seifa5			1			
Prm11	ra3				2		
Prm12	ra3				3		
Prm13	ra3				1		
Prm14	birth_cohort					2	
Prm15	birth_cohort					3	
Prm16	birth_cohort					4	
Prm17	birth_cohort					1	
Prm18	danger_harm						1
Prm19	danger_harm						0

Estimated Correlation Matrix

	Prm1	Prm2	Prm4	Prm6	Prm7	Prm8	Prm9	Prm11	Prm12	Prm14	Prm15	Prm16	Prm18
Prm1	1.0000	-0.4198	-0.2749	-0.3385	-0.3120	-0.3360	-0.3608	-0.2762	-0.1946	-0.6529	-0.6323	-0.4473	-0.0142
Prm2	-0.4198	1.0000	0.1035	0.0083	-0.0103	-0.0022	-0.0019	0.0187	0.0270	0.0264	0.0606	0.0607	-0.0543
Prm4	-0.2749	0.1035	1.0000	0.1167	0.1644	0.1913	0.2018	-0.0729	-0.4859	0.0116	-0.0033	-0.0232	0.1059
Prm6	-0.3385	0.0083	0.1167	1.0000	0.3634	0.3531	0.3356	0.1050	0.0176	0.0076	-0.0078	-0.0053	-0.0244
Prm7	-0.3120	-0.0103	0.1644	0.3634	1.0000	0.3140	0.3070	0.0820	0.0393	0.0042	0.0104	0.0146	0.0077
Prm8	-0.3360	-0.0022	0.1913	0.3531	0.3140	1.0000	0.3273	0.1607	0.0972	0.0126	0.0036	0.0122	-0.0530
Prm9	-0.3608	-0.0019	0.2018	0.3356	0.3070	0.3273	1.0000	0.2475	0.1665	0.0046	0.0119	0.0237	-0.0101
Prm11	-0.2762	0.0187	-0.0729	0.1050	0.0820	0.1607	0.2475	1.0000	0.3732	0.0092	0.0072	-0.0029	-0.0819
Prm12	-0.1946	0.0270	-0.4859	0.0176	0.0393	0.0972	0.1665	0.3732	1.0000	0.0120	0.0280	0.0087	-0.0664
Prm14	-0.6529	0.0264	0.0116	0.0076	0.0042	0.0126	0.0046	0.0092	0.0120	1.0000	0.7515	0.5359	-0.0212

Estimated Correlation Matrix													
	**Prm1**	**Prm2**	**Prm4**	**Prm6**	**Prm7**	**Prm8**	**Prm9**	**Prm11**	**Prm12**	**Prm14**	**Prm15**	**Prm16**	**Prm18**
Prm15	-0.6323	0.0606	-0.0033	-0.0078	0.0104	0.0036	0.0119	0.0072	0.0280	0.7515	1.0000	0.5202	-0.0673
Prm16	-0.4473	0.0607	-0.0232	-0.0053	0.0146	0.0122	0.0237	-0.0029	0.0087	0.5359	0.5202	1.0000	-0.1305
Prm18	-0.0142	-0.0543	0.1059	-0.0244	0.0077	-0.0530	-0.0101	-0.0819	-0.0664	-0.0212	-0.0673	-0.1305	1.0000

Damage

Parameter Information							
**Parameter**	**Effect**	**gender**	**child_gosr**	**seifa5**	**ra3**	**birth_cohort**	**damage**
Prm1	Intercept						
Prm2	gender	1					
Prm3	gender	0					
Prm4	child_gosr		1				
Prm5	child_gosr		0				
Prm6	seifa5			2			
Prm7	seifa5			3			
Prm8	seifa5			4			
Prm9	seifa5			5			
Prm10	seifa5			1			
Prm11	ra3				2		
Prm12	ra3				3		
Prm13	ra3				1		
Prm14	birth_cohort					2	
Prm15	birth_cohort					3	
Prm16	birth_cohort					4	
Prm17	birth_cohort					1	
Prm18	damage						1
Prm19	damage						0

Estimated Correlation Matrix													
	**Prm1**	**Prm2**	**Prm4**	**Prm6**	**Prm7**	**Prm8**	**Prm9**	**Prm11**	**Prm12**	**Prm14**	**Prm15**	**Prm16**	**Prm18**
Prm1	1.0000	-0.4274	-0.2599	-0.3396	-0.3062	-0.3303	-0.3531	-0.2820	-0.2032	-0.6540	-0.6329	-0.4649	-0.0556
Prm2	-0.4274	1.0000	0.1068	0.0101	-0.0123	-0.0154	-0.0079	0.0168	0.0332	0.0337	0.0612	0.0661	0.0112
Prm4	-0.2599	0.1068	1.0000	0.1132	0.1593	0.1867	0.1997	-0.0732	-0.4911	0.0041	-0.0093	-0.0191	0.0256
Prm6	-0.3396	0.0101	0.1132	1.0000	0.3624	0.3511	0.3347	0.1102	0.0156	0.0083	-0.0096	0.0028	0.0005

Estimated Correlation Matrix													
	**Prm1**	**Prm2**	**Prm4**	**Prm6**	**Prm7**	**Prm8**	**Prm9**	**Prm11**	**Prm12**	**Prm14**	**Prm15**	**Prm16**	**Prm18**
Prm7	-0.3062	-0.0123	0.1593	0.3624	1.0000	0.3126	0.3049	0.0788	0.0382	0.0044	0.0055	0.0177	-0.0011
Prm8	-0.3303	-0.0154	0.1867	0.3511	0.3126	1.0000	0.3261	0.1623	0.0952	0.0094	0.0013	0.0067	0.0053
Prm9	-0.3531	-0.0079	0.1997	0.3347	0.3049	0.3261	1.0000	0.2462	0.1652	-0.0010	0.0054	0.0189	0.0096
Prm11	-0.2820	0.0168	-0.0732	0.1102	0.0788	0.1623	0.2462	1.0000	0.3720	0.0102	0.0045	0.0061	-0.0075
Prm12	-0.2032	0.0332	-0.4911	0.0156	0.0382	0.0952	0.1652	0.3720	1.0000	0.0151	0.0286	0.0214	0.0093
Prm14	-0.6540	0.0337	0.0041	0.0083	0.0044	0.0094	-0.0010	0.0102	0.0151	1.0000	0.7522	0.5388	0.0026
Prm15	-0.6329	0.0612	-0.0093	-0.0096	0.0055	0.0013	0.0054	0.0045	0.0286	0.7522	1.0000	0.5180	0.0129
Prm16	-0.4649	0.0661	-0.0191	0.0028	0.0177	0.0067	0.0189	0.0061	0.0214	0.5388	0.5180	1.0000	0.0190
Prm18	-0.0556	0.0112	0.0256	0.0005	-0.0011	0.0053	0.0096	-0.0075	0.0093	0.0026	0.0129	0.0190	1.0000

Criminal damage

Parameter Information							
**Parameter**	**Effect**	**gender**	**child_gosr**	**seifa5**	**ra3**	**birth_cohort**	**crim_damage**
Prm1	Intercept						
Prm2	gender	1					
Prm3	gender	0					
Prm4	child_gosr		1				
Prm5	child_gosr		0				
Prm6	seifa5			2			
Prm7	seifa5			3			
Prm8	seifa5			4			
Prm9	seifa5			5			
Prm10	seifa5			1			
Prm11	ra3				2		
Prm12	ra3				3		
Prm13	ra3				1		
Prm14	birth_cohort					2	
Prm15	birth_cohort					3	
Prm16	birth_cohort					4	
Prm17	birth_cohort					1	
Prm18	crim_damage						1
Prm19	crim_damage						0

Estimated Correlation Matrix													
	**Prm1**	**Prm2**	**Prm4**	**Prm6**	**Prm7**	**Prm8**	**Prm9**	**Prm11**	**Prm12**	**Prm14**	**Prm15**	**Prm16**	**Prm18**
Prm1	1.0000	-0.4269	-0.2599	-0.3401	-0.3083	-0.3281	-0.3537	-0.2820	-0.2049	-0.6534	-0.6314	-0.4638	-0.0529
Prm2	-0.4269	1.0000	0.1042	0.0090	-0.0111	-0.0167	-0.0068	0.0190	0.0356	0.0341	0.0596	0.0660	0.0020
Prm4	-0.2599	0.1042	1.0000	0.1148	0.1627	0.1869	0.2016	-0.0727	-0.4897	0.0048	-0.0092	-0.0200	0.0100
Prm6	-0.3401	0.0090	0.1148	1.0000	0.3631	0.3518	0.3358	0.1133	0.0159	0.0077	-0.0107	0.0015	-0.0012
Prm7	-0.3083	-0.0111	0.1627	0.3631	1.0000	0.3135	0.3060	0.0808	0.0373	0.0042	0.0050	0.0178	0.0068
Prm8	-0.3281	-0.0167	0.1869	0.3518	0.3135	1.0000	0.3270	0.1637	0.0969	0.0045	-0.0021	0.0041	0.0078
Prm9	-0.3537	-0.0068	0.2016	0.3358	0.3060	0.3270	1.0000	0.2466	0.1651	-0.0023	0.0035	0.0166	0.0152
Prm11	-0.2820	0.0190	-0.0727	0.1133	0.0808	0.1637	0.2466	1.0000	0.3724	0.0063	0.0007	0.0042	0.0054
Prm12	-0.2049	0.0356	-0.4897	0.0159	0.0373	0.0969	0.1651	0.3724	1.0000	0.0143	0.0285	0.0217	0.0226
Prm14	-0.6534	0.0341	0.0048	0.0077	0.0042	0.0045	-0.0023	0.0063	0.0143	1.0000	0.7522	0.5389	0.0076
Prm15	-0.6314	0.0596	-0.0092	-0.0107	0.0050	-0.0021	0.0035	0.0007	0.0285	0.7522	1.0000	0.5179	0.0122
Prm16	-0.4638	0.0660	-0.0200	0.0015	0.0178	0.0041	0.0166	0.0042	0.0217	0.5389	0.5179	1.0000	0.0159
Prm18	-0.0529	0.0020	0.0100	-0.0012	0.0068	0.0078	0.0152	0.0054	0.0226	0.0076	0.0122	0.0159	1.0000

Deprivation of liberty

Parameter Information							
**Parameter**	**Effect**	**gender**	**child_gosr**	**seifa5**	**ra3**	**birth_cohort**	**deprivation**
Prm1	Intercept						
Prm2	gender	1					
Prm3	gender	0					
Prm4	child_gosr		1				
Prm5	child_gosr		0				
Prm6	seifa5			2			
Prm7	seifa5			3			
Prm8	seifa5			4			
Prm9	seifa5			5			
Prm10	seifa5			1			
Prm11	ra3				2		
Prm12	ra3				3		
Prm13	ra3				1		
Prm14	birth_cohort					2	
Prm15	birth_cohort					3	
Prm16	birth_cohort					4	
Prm17	birth_cohort					1	

Parameter Information							
Parameter	Effect	gender	child_gosr	seifa5	ra3	birth_cohort	deprivation
Prm18	deprivation						1
Prm19	deprivation						0

Estimated Correlation Matrix

	Prm1	Prm2	Prm4	Prm6	Prm7	Prm8	Prm9	Prm11	Prm12	Prm14	Prm15	Prm16	Prm18
Prm1	1.0000	-0.4254	-0.2578	-0.3403	-0.3079	-0.3286	-0.3537	-0.2827	-0.2040	-0.6544	-0.6320	-0.4637	-0.0645
Prm2	-0.4254	1.0000	0.1020	0.0100	-0.0117	-0.0181	-0.0069	0.0186	0.0339	0.0327	0.0583	0.0631	0.0241
Prm4	-0.2578	0.1020	1.0000	0.1139	0.1623	0.1865	0.2004	-0.0722	-0.4874	0.0052	-0.0109	-0.0201	-0.0147
Prm6	-0.3403	0.0100	0.1139	1.0000	0.3631	0.3516	0.3358	0.1138	0.0176	0.0072	-0.0113	0.0033	-0.0063
Prm7	-0.3079	-0.0117	0.1623	0.3631	1.0000	0.3133	0.3058	0.0816	0.0374	0.0045	0.0048	0.0175	0.0035
Prm8	-0.3286	-0.0181	0.1865	0.3516	0.3133	1.0000	0.3263	0.1625	0.0965	0.0073	-0.0009	0.0061	0.0061
Prm9	-0.3537	-0.0069	0.2004	0.3358	0.3058	0.3263	1.0000	0.2471	0.1664	-0.0017	0.0039	0.0174	0.0128
Prm11	-0.2827	0.0186	-0.0722	0.1138	0.0816	0.1625	0.2471	1.0000	0.3724	0.0071	0.0018	0.0051	0.0081
Prm12	-0.2040	0.0339	-0.4874	0.0176	0.0374	0.0965	0.1664	0.3724	1.0000	0.0136	0.0279	0.0186	0.0083
Prm14	-0.6544	0.0327	0.0052	0.0072	0.0045	0.0073	-0.0017	0.0071	0.0136	1.0000	0.7523	0.5391	0.0246
Prm15	-0.6320	0.0583	-0.0109	-0.0113	0.0048	-0.0009	0.0039	0.0018	0.0279	0.7523	1.0000	0.5181	0.0404
Prm16	-0.4637	0.0631	-0.0201	0.0033	0.0175	0.0061	0.0174	0.0051	0.0186	0.5391	0.5181	1.0000	0.0289
Prm18	-0.0645	0.0241	-0.0147	-0.0063	0.0035	0.0061	0.0128	0.0081	0.0083	0.0246	0.0404	0.0289	1.0000

Act intended to cause grievous bodily harm or prevent arrest

Parameter Information							
Parameter	Effect	gender	child_gosr	seifa5	ra3	birth_cohort	act_arrest
Prm1	Intercept						
Prm2	gender	1					
Prm3	gender	0					
Prm4	child_gosr		1				
Prm5	child_gosr		0				
Prm6	seifa5			2			
Prm7	seifa5			3			
Prm8	seifa5			4			
Prm9	seifa5			5			
Prm10	seifa5			1			
Prm11	ra3				2		

Parameter Information

Parameter	Effect	gender	child_gosr	seifa5	ra3	birth_cohort	act_arrest
Prm12	ra3				3		
Prm13	ra3				1		
Prm14	birth_cohort					2	
Prm15	birth_cohort					3	
Prm16	birth_cohort					4	
Prm17	birth_cohort					1	
Prm18	act_arrest						1
Prm19	act_arrest						0

Estimated Correlation Matrix

	Prm1	Prm2	Prm4	Prm6	Prm7	Prm8	Prm9	Prm11	Prm12	Prm14	Prm15	Prm16	Prm18
Prm1	1.0000	-0.4238	-0.2598	-0.3392	-0.3075	-0.3294	-0.3540	-0.2852	-0.2065	-0.6529	-0.6312	-0.4619	-0.0034
Prm2	-0.4238	1.0000	0.1036	0.0103	-0.0119	-0.0176	-0.0061	0.0178	0.0338	0.0315	0.0577	0.0621	-0.0667
Prm4	-0.2598	0.1036	1.0000	0.1137	0.1638	0.1899	0.2010	-0.0727	-0.4861	0.0061	-0.0106	-0.0209	-0.0196
Prm6	-0.3392	0.0103	0.1137	1.0000	0.3630	0.3516	0.3354	0.1126	0.0159	0.0058	-0.0124	0.0010	-0.0107
Prm7	-0.3075	-0.0119	0.1638	0.3630	1.0000	0.3140	0.3062	0.0815	0.0372	0.0026	0.0036	0.0160	-0.0023
Prm8	-0.3294	-0.0176	0.1899	0.3516	0.3140	1.0000	0.3271	0.1619	0.0943	0.0069	0.0010	0.0056	-0.0217
Prm9	-0.3540	-0.0061	0.2010	0.3354	0.3062	0.3271	1.0000	0.2464	0.1660	-0.0028	0.0050	0.0164	-0.0074
Prm11	-0.2852	0.0178	-0.0727	0.1126	0.0815	0.1619	0.2464	1.0000	0.3746	0.0072	0.0035	0.0040	0.0543
Prm12	-0.2065	0.0338	-0.4861	0.0159	0.0372	0.0943	0.1660	0.3746	1.0000	0.0133	0.0298	0.0196	0.0506
Prm14	-0.6529	0.0315	0.0061	0.0058	0.0026	0.0069	-0.0028	0.0072	0.0133	1.0000	0.7522	0.5383	-0.0199
Prm15	-0.6312	0.0577	-0.0106	-0.0124	0.0036	0.0010	0.0050	0.0035	0.0298	0.7522	1.0000	0.5173	-0.0243
Prm16	-0.4619	0.0621	-0.0209	0.0010	0.0160	0.0056	0.0164	0.0040	0.0196	0.5383	0.5173	1.0000	0.0075
Prm18	-0.0034	-0.0667	-0.0196	-0.0107	-0.0023	-0.0217	-0.0074	0.0543	0.0506	-0.0199	-0.0243	0.0075	1.0000

Stealing

Parameter Information

Parameter	Effect	gender	child_gosr	seifa5	ra3	birth_cohort	steal
Prm1	Intercept						
Prm2	gender	1					
Prm3	gender	0					
Prm4	child_gosr		1				
Prm5	child_gosr		0				

Parameter Information							
Parameter	Effect	gender	child_gos	rseifa5	ra3	birth_cohort	steal
Prm6	seifa5			2			
Prm7	seifa5			3			
Prm8	seifa5			4			
Prm9	seifa5			5			
Prm10	seifa5			1			
Prm11	ra3				2		
Prm12	ra3				3		
Prm13	ra3				1		
Prm14	birth_cohort					2	
Prm15	birth_cohort					3	
Prm16	birth_cohort					4	
Prm17	birth_cohort					1	
Prm18	steal						1
Prm19	steal						0

Estimated Correlation Matrix

	Prm1	Prm2	Prm4	Prm6	Prm7	Prm8	Prm9	Prm11	Prm12	Prm14	Prm15	Prm16	Prm18
Prm1	1.0000	-0.4273	-0.2622	-0.3409	-0.3066	-0.3270	-0.3523	-0.2812	-0.2009	-0.6547	-0.6314	-0.4620	-0.0352
Prm2	-0.4273	1.0000	0.1079	0.0104	-0.0120	-0.0206	-0.0095	0.0167	0.0326	0.0347	0.0606	0.0643	0.0022
Prm4	-0.2622	0.1079	1.0000	0.1131	0.1615	0.1856	0.1982	-0.0738	-0.4921	0.0086	-0.0075	-0.0177	0.0137
Prm6	-0.3409	0.0104	0.1131	1.0000	0.3630	0.3518	0.3361	0.1153	0.0183	0.0074	-0.0120	0.0019	0.0030
Prm7	-0.3066	-0.0120	0.1615	0.3630	1.0000	0.3128	0.3050	0.0805	0.0351	0.0045	0.0053	0.0153	-0.0062
Prm8	-0.3270	-0.0206	0.1856	0.3518	0.3128	1.0000	0.3259	0.1621	0.0951	0.0070	-0.0012	0.0054	0.0031
Prm9	-0.3523	-0.0095	0.1982	0.3361	0.3050	0.3259	1.0000	0.2468	0.1652	-0.0009	0.0050	0.0187	-0.0074
Prm11	-0.2812	0.0167	-0.0738	0.1153	0.0805	0.1621	0.2468	1.0000	0.3723	0.0065	0.0013	0.0033	0.0023
Prm12	-0.2009	0.0326	-0.4921	0.0183	0.0351	0.0951	0.1652	0.3723	1.0000	0.0119	0.0259	0.0177	0.0098
Prm14	-0.6547	0.0347	0.0086	0.0074	0.0045	0.0070	-0.0009	0.0065	0.0119	1.0000	0.7521	0.5387	0.0010
Prm15	-0.6314	0.0606	-0.0075	-0.0120	0.0053	-0.0012	0.0050	0.0013	0.0259	0.7521	1.0000	0.5179	-0.0099
Prm16	-0.4620	0.0643	-0.0177	0.0019	0.0153	0.0054	0.0187	0.0033	0.0177	0.5387	0.5179	1.0000	-0.0213
Prm18	-0.0352	0.0022	0.0137	0.0030	-0.0062	0.0031	-0.0074	0.0023	0.0098	0.0010	-0.0099	-0.0213	1.0000

Robbery in circumstances of aggravation

Parameter Information

Parameter	Effect	gender	child_gosr	seifa5	ra3	birth_cohort	rob
Prm1	Intercept						
Prm2	gender	1					
Prm3	gender	0					
Prm4	child_gosr		1				
Prm5	child_gosr		0				
Prm6	seifa5			2			
Prm7	seifa5			3			
Prm8	seifa5			4			
Prm9	seifa5			5			
Prm10	seifa5			1			
Prm11	ra3				2		
Prm12	ra3				3		
Prm13	ra3				1		
Prm14	birth_cohort					2	
Prm15	birth_cohort					3	
Prm16	birth_cohort					4	
Prm17	birth_cohort					1	
Prm18	rob						1
Prm19	rob						0

Estimated Correlation Matrix

	Prm1	Prm2	Prm4	Prm6	Prm7	Prm8	Prm9	Prm11	Prm12	Prm14	Prm15	Prm16	Prm18
Prm1	1.0000	-0.4243	-0.2585	-0.3417	-0.3084	-0.3293	-0.3533	-0.2824	-0.2026	-0.6544	-0.6310	-0.4622	-0.0135
Prm2	-0.4243	1.0000	0.1025	0.0098	-0.0121	-0.0179	-0.0070	0.0161	0.0299	0.0332	0.0604	0.0663	-0.0384
Prm4	-0.2585	0.1025	1.0000	0.1132	0.1615	0.1864	0.2004	-0.0734	-0.4891	0.0053	-0.0099	-0.0195	-0.0041
Prm6	-0.3417	0.0098	0.1132	1.0000	0.3632	0.3518	0.3360	0.1140	0.0181	0.0081	-0.0105	0.0038	-0.0009
Prm7	-0.3084	-0.0121	0.1615	0.3632	1.0000	0.3134	0.3059	0.0820	0.0382	0.0045	0.0050	0.0168	-0.0009
Prm8	-0.3293	-0.0179	0.1864	0.3518	0.3134	1.0000	0.3262	0.1625	0.0962	0.0077	-0.0008	0.0055	0.0014
Prm9	-0.3533	-0.0070	0.2004	0.3360	0.3059	0.3262	1.0000	0.2459	0.1650	-0.0017	0.0038	0.0166	-0.0111
Prm11	-0.2824	0.0161	-0.0734	0.1140	0.0820	0.1625	0.2459	1.0000	0.3735	0.0067	0.0004	0.0018	0.0329
Prm12	-0.2026	0.0299	-0.4891	0.0181	0.0382	0.0962	0.1650	0.3735	1.0000	0.0125	0.0248	0.0145	0.0468
Prm14	-0.6544	0.0332	0.0053	0.0081	0.0045	0.0077	-0.0017	0.0067	0.0125	1.0000	0.7521	0.5388	-0.0116
Prm15	-0.6310	0.0604	-0.0099	-0.0105	0.0050	-0.0008	0.0038	0.0004	0.0248	0.7521	1.0000	0.5183	-0.0337
Prm16	-0.4622	0.0663	-0.0195	0.0038	0.0168	0.0055	0.0166	0.0018	0.0145	0.5388	0.5183	1.0000	-0.0376
Prm18	-0.0135	-0.0384	-0.0041	-0.0009	-0.0009	0.0014	-0.0111	0.0329	0.0468	-0.0116	-0.0337	-0.0376	1.0000

Dangerous driving causing death

Parameter Information

Parameter	Effect	gender	child_gosr	seifa5	ra3	birth_cohort	danger_death
Prm1	Intercept						
Prm2	gender	1					
Prm3	gender	0					
Prm4	child_gosr		1				
Prm5	child_gosr		0				
Prm6	seifa5			2			
Prm7	seifa5			3			
Prm8	seifa5			4			
Prm9	seifa5			5			
Prm10	seifa5			1			
Prm11	ra3				2		
Prm12	ra3				3		
Prm13	ra3				1		
Prm14	birth_cohort					2	
Prm15	birth_cohort					3	
Prm16	birth_cohort					4	
Prm17	birth_cohort					1	
Prm18	danger_death						1
Prm19	danger_death						0

Estimated Correlation Matrix

	Prm1	Prm2	Prm4	Prm6	Prm7	Prm8	Prm9	Prm11	Prm12	Prm14	Prm15	Prm16	Prm18
Prm1	1.0000	-0.4224	-0.2592	-0.3414	-0.3118	-0.3303	-0.3530	-0.2859	-0.2021	-0.6534	-0.6319	-0.4609	-0.0004
Prm2	-0.4224	1.0000	0.1014	0.0088	-0.0097	-0.0154	-0.0102	0.0215	0.0304	0.0310	0.0561	0.0595	-0.0299
Prm4	-0.2592	0.1014	1.0000	0.1136	0.1629	0.1878	0.1968	-0.0715	-0.4900	0.0056	-0.0084	-0.0213	0.0070
Prm6	-0.3414	0.0088	0.1136	1.0000	0.3640	0.3522	0.3368	0.1178	0.0190	0.0071	-0.0121	0.0016	-0.0121
Prm7	-0.3118	-0.0097	0.1629	0.3640	1.0000	0.3141	0.3084	0.0867	0.0393	0.0065	0.0077	0.0199	-0.0437
Prm8	-0.3303	-0.0154	0.1878	0.3522	0.3141	1.0000	0.3259	0.1603	0.0962	0.0064	-0.0004	0.0056	0.0002
Prm9	-0.3530	-0.0102	0.1968	0.3368	0.3084	0.3259	1.0000	0.2491	0.1687	-0.0026	0.0070	0.0176	-0.0375
Prm11	-0.2859	0.0215	-0.0715	0.1178	0.0867	0.1603	0.2491	1.0000	0.3722	0.0074	0.0018	0.0051	-0.0011
Prm12	-0.2021	0.0304	-0.4900	0.0190	0.0393	0.0962	0.1687	0.3722	1.0000	0.0123	0.0277	0.0162	-0.0088
Prm14	-0.6534	0.0310	0.0056	0.0071	0.0065	0.0064	-0.0026	0.0074	0.0123	1.0000	0.7520	0.5386	-0.0198

Estimated Correlation Matrix

	Prm1	Prm2	Prm4	Prm6	Prm7	Prm8	Prm9	Prm11	Prm12	Prm14	Prm15	Prm16	Prm18
Prm15	-0.6319	0.0561	-0.0084	-0.0121	0.0077	-0.0004	0.0070	0.0018	0.0277	0.7520	1.0000	0.5175	-0.0077
Prm16	-0.4609	0.0595	-0.0213	0.0016	0.0199	0.0056	0.0176	0.0051	0.0162	0.5386	0.5175	1.0000	-0.0057
Prm18	-0.0004	-0.0299	0.0070	-0.0121	-0.0437	0.0002	-0.0375	-0.0011	-0.0088	-0.0198	-0.0077	-0.0057	1.0000

Breach violence restraining order

Parameter Information

Parameter	Effect	gender	child_gosr	seifa5	ra3	birth_cohort	breach_violence
Prm1	Intercept						
Prm2	gender	1					
Prm3	gender	0					
Prm4	child_gosr		1				
Prm5	child_gosr		0				
Prm6	seifa5			2			
Prm7	seifa5			3			
Prm8	seifa5			4			
Prm9	seifa5			5			
Prm10	seifa5			1			
Prm11	ra3				2		
Prm12	ra3				3		
Prm13	ra3				1		
Prm14	birth_cohort					2	
Prm15	birth_cohort					3	
Prm16	birth_cohort					4	
Prm17	birth_cohort					1	
Prm18	breach_violence						1
Prm19	breach_violence						0

Estimated Correlation Matrix

	Prm1	Prm2	Prm4	Prm6	Prm7	Prm8	Prm9	Prm11	Prm12	Prm14	Prm15	Prm16	Prm18
Prm1	1.0000	-0.4254	-0.2560	-0.3409	-0.3081	-0.3308	-0.3545	-0.2845	-0.2050	-0.6527	-0.6311	-0.4629	-0.0506
Prm2	-0.4254	1.0000	0.1016	0.0094	-0.0113	-0.0169	-0.0057	0.0193	0.0351	0.0303	0.0575	0.0631	0.0308
Prm4	-0.2560	0.1016	1.0000	0.1151	0.1622	0.1861	0.2001	-0.0731	-0.4855	0.0032	-0.0139	-0.0230	-0.0345
Prm6	-0.3409	0.0094	0.1151	1.0000	0.3636	0.3525	0.3369	0.1143	0.0198	0.0056	-0.0121	0.0027	-0.0020

Estimated Correlation Matrix

	Prm1	Prm2	Prm4	Prm6	Prm7	Prm8	Prm9	Prm11	Prm12	Prm14	Prm15	Prm16	Prm18
Prm7	-0.3081	-0.0113	0.1622	0.3636	1.0000	0.3133	0.3058	0.0814	0.0369	0.0032	0.0052	0.0172	0.0093
Prm8	-0.3308	-0.0169	0.1861	0.3525	0.3133	1.0000	0.3267	0.1634	0.0977	0.0085	0.0005	0.0064	0.0088
Prm9	-0.3545	-0.0057	0.2001	0.3369	0.3058	0.3267	1.0000	0.2476	0.1670	-0.0030	0.0040	0.0170	0.0147
Prm11	-0.2845	0.0193	-0.0731	0.1143	0.0814	0.1634	0.2476	1.0000	0.3732	0.0080	0.0038	0.0064	0.0123
Prm12	-0.2050	0.0351	-0.4855	0.0198	0.0369	0.0977	0.1670	0.3732	1.0000	0.0129	0.0278	0.0186	-0.0020
Prm14	-0.6527	0.0303	0.0032	0.0056	0.0032	0.0085	-0.0030	0.0080	0.0129	1.0000	0.7521	0.5388	0.0161
Prm15	-0.6311	0.0575	-0.0139	-0.0121	0.0052	0.0005	0.0040	0.0038	0.0278	0.7521	1.0000	0.5180	0.0274
Prm16	-0.4629	0.0631	-0.0230	0.0027	0.0172	0.0064	0.0170	0.0064	0.0186	0.5388	0.5180	1.0000	0.0237
Prm18	-0.0506	0.0308	-0.0345	-0.0020	0.0093	0.0088	0.0147	0.0123	-0.0020	0.0161	0.0274	0.0237	1.0000

Threats to harm, take control of conveyance or building

Parameter Information

Parameter	Effect	gender	child_gosr	seifa5	ra3	birth_cohort	threat_build
Prm1	Intercept						
Prm2	gender	1					
Prm3	gender	0					
Prm4	child_gosr		1				
Prm5	child_gosr		0				
Prm6	seifa5			2			
Prm7	seifa5			3			
Prm8	seifa5			4			
Prm9	seifa5			5			
Prm10	seifa5			1			
Prm11	ra3				2		
Prm12	ra3				3		
Prm13	ra3				1		
Prm14	birth_cohort					2	
Prm15	birth_cohort					3	
Prm16	birth_cohort					4	
Prm17	birth_cohort					1	
Prm18	threat_build						1
Prm19	threat_build						0

Estimated Correlation Matrix													
	**Prm1**	**Prm2**	**Prm4**	**Prm6**	**Prm7**	**Prm8**	**Prm9**	**Prm11**	**Prm12**	**Prm14**	**Prm15**	**Prm16**	**Prm18**
Prm1	1.0000	-0.4252	-0.2593	-0.3423	-0.3085	-0.3298	-0.3548	-0.2827	-0.2048	-0.6534	-0.6305	-0.4629	-0.0441
Prm2	-0.4252	1.0000	0.1039	0.0107	-0.0115	-0.0172	-0.0073	0.0188	0.0343	0.0319	0.0569	0.0628	0.0072
Prm4	-0.2593	0.1039	1.0000	0.1152	0.1626	0.1887	0.2011	-0.0731	-0.4882	0.0048	-0.0112	-0.0215	-0.0016
Prm6	-0.3423	0.0107	0.1152	1.0000	0.3635	0.3523	0.3367	0.1139	0.0182	0.0074	-0.0111	0.0037	0.0135
Prm7	-0.3085	-0.0115	0.1626	0.3635	1.0000	0.3137	0.3061	0.0809	0.0379	0.0040	0.0050	0.0174	0.0080
Prm8	-0.3298	-0.0172	0.1887	0.3523	0.3137	1.0000	0.3268	0.1612	0.0954	0.0073	-0.0007	0.0057	0.0129
Prm9	-0.3548	-0.0073	0.2011	0.3367	0.3061	0.3268	1.0000	0.2463	0.1660	-0.0012	0.0044	0.0180	0.0222
Prm11	-0.2827	0.0188	-0.0731	0.1139	0.0809	0.1612	0.2463	1.0000	0.3726	0.0078	0.0026	0.0054	-0.0012
Prm12	-0.2048	0.0343	-0.4882	0.0182	0.0379	0.0954	0.1660	0.3726	1.0000	0.0141	0.0288	0.0206	0.0128
Prm14	-0.6534	0.0319	0.0048	0.0074	0.0040	0.0073	-0.0012	0.0078	0.0141	1.0000	0.7521	0.5388	0.0020
Prm15	-0.6305	0.0569	-0.0112	-0.0111	0.0050	-0.0007	0.0044	0.0026	0.0288	0.7521	1.0000	0.5177	0.0034
Prm16	-0.4629	0.0628	-0.0215	0.0037	0.0174	0.0057	0.0180	0.0054	0.0206	0.5388	0.5177	1.0000	0.0081
Prm18	-0.0441	0.0072	-0.0016	0.0135	0.0080	0.0129	0.0222	-0.0012	0.0128	0.0020	0.0034	0.0081	1.0000

Burglary and commit

Parameter Information							
**Parameter**	**Effect**	**gender**	**child_gosr**	**seifa5**	**ra3**	**birth_cohort**	**burg_commit**
Prm1	Intercept						
Prm2	gender	1					
Prm3	gender	0					
Prm4	child_gosr		1				
Prm5	child_gosr		0				
Prm6	seifa5			2			
Prm7	seifa5			3			
Prm8	seifa5			4			
Prm9	seifa5			5			
Prm10	seifa5			1			
Prm11	ra3				2		
Prm12	ra3				3		
Prm13	ra3				1		
Prm14	birth_cohort					2	
Prm15	birth_cohort					3	
Prm16	birth_cohort					4	
Prm17	birth_cohort					1	

Parameter Information							
Parameter	Effect	gender	child_gosr	seifa5	ra3	birth_cohort	burg_commit
Prm18	burg_commit						1
Prm19	burg_commit						0

Estimated Correlation Matrix													
	**Prm1**	**Prm2**	**Prm4**	**Prm6**	**Prm7**	**Prm8**	**Prm9**	**Prm11**	**Prm12**	**Prm14**	**Prm15**	**Prm16**	**Prm18**
Prm1	1.0000	-0.4260	-0.2605	-0.3406	-0.3084	-0.3294	-0.3538	-0.2830	-0.2025	-0.6543	-0.6317	-0.4636	-0.0296
Prm2	-0.4260	1.0000	0.1050	0.0104	-0.0114	-0.0178	-0.0074	0.0195	0.0329	0.0332	0.0590	0.0636	-0.0041
Prm4	-0.2605	0.1050	1.0000	0.1143	0.1628	0.1866	0.2002	-0.0728	-0.4890	0.0065	-0.0091	-0.0191	0.0014
Prm6	-0.3406	0.0104	0.1143	1.0000	0.3633	0.3517	0.3360	0.1138	0.0173	0.0071	-0.0116	0.0030	-0.0052
Prm7	-0.3084	-0.0114	0.1628	0.3633	1.0000	0.3135	0.3060	0.0819	0.0374	0.0046	0.0050	0.0174	-0.0038
Prm8	-0.3294	-0.0178	0.1866	0.3517	0.3135	1.0000	0.3263	0.1622	0.0959	0.0080	-0.0001	0.0064	0.0098
Prm9	-0.3538	-0.0074	0.2002	0.3360	0.3060	0.3263	1.0000	0.2473	0.1659	-0.0013	0.0045	0.0178	0.0038
Prm11	-0.2830	0.0195	-0.0728	0.1138	0.0819	0.1622	0.2473	1.0000	0.3727	0.0077	0.0022	0.0047	-0.0089
Prm12	-0.2025	0.0329	-0.4890	0.0173	0.0374	0.0959	0.1659	0.3727	1.0000	0.0129	0.0274	0.0194	-0.0052
Prm14	-0.6543	0.0332	0.0065	0.0071	0.0046	0.0080	-0.0013	0.0077	0.0129	1.0000	0.7522	0.5388	0.0040
Prm15	-0.6317	0.0590	-0.0091	-0.0116	0.0050	-0.0001	0.0045	0.0022	0.0274	0.7522	1.0000	0.5178	0.0106
Prm16	-0.4636	0.0636	-0.0191	0.0030	0.0174	0.0064	0.0178	0.0047	0.0194	0.5388	0.5178	1.0000	0.0136
Prm18	-0.0296	-0.0041	0.0014	-0.0052	-0.0038	0.0098	0.0038	-0.0089	-0.0052	0.0040	0.0106	0.0136	1.0000

Breach police restraining order

Parameter Information							
Parameter	Effect	gender	child_gosr	seifa5	ra3	birth_cohort	breach_police
Prm1	Intercept						
Prm2	gender	1					
Prm3	gender	0					
Prm4	child_gosr		1				
Prm5	child_gosr		0				
Prm6	seifa5			2			
Prm7	seifa5			3			
Prm8	seifa5			4			
Prm9	seifa5			5			
Prm10	seifa5			1			
Prm11	ra3				2		

Parameter	Effect	gender	child_gosr	seifa5	ra3	birth_cohort	breach_police
Prm12	ra3				3		
Prm13	ra3				1		
Prm14	birth_cohort					2	
Prm15	birth_cohort					3	
Prm16	birth_cohort					4	
Prm17	birth_cohort					1	
Prm18	breach_police						1
Prm19	breach_police						0

Parameter Information

Estimated Correlation Matrix

	Prm1	Prm2	Prm4	Prm6	Prm7	Prm8	Prm9	Prm11	Prm12	Prm14	Prm15	Prm16	Prm18
Prm1	1.0000	-0.4254	-0.2597	-0.3429	-0.3093	-0.3295	-0.3543	-0.2829	-0.2061	-0.6538	-0.6299	-0.4624	-0.0233
Prm2	-0.4254	1.0000	0.1028	0.0118	-0.0090	-0.0160	-0.0063	0.0177	0.0353	0.0311	0.0552	0.0617	0.0265
Prm4	-0.2597	0.1028	1.0000	0.1142	0.1628	0.1874	0.2002	-0.0712	-0.4848	0.0064	-0.0088	-0.0205	-0.0295
Prm6	-0.3429	0.0118	0.1142	1.0000	0.3635	0.3521	0.3365	0.1157	0.0184	0.0084	-0.0106	0.0034	0.0037
Prm7	-0.3093	-0.0090	0.1628	0.3635	1.0000	0.3135	0.3061	0.0834	0.0373	0.0042	0.0033	0.0176	0.0105
Prm8	-0.3295	-0.0160	0.1874	0.3521	0.3135	1.0000	0.3263	0.1629	0.0960	0.0073	-0.0017	0.0056	0.0037
Prm9	-0.3543	-0.0063	0.2002	0.3365	0.3061	0.3263	1.0000	0.2480	0.1671	-0.0012	0.0031	0.0168	0.0072
Prm11	-0.2829	0.0177	-0.0712	0.1157	0.0834	0.1629	0.2480	1.0000	0.3724	0.0072	0.0013	0.0045	-0.0067
Prm12	-0.2061	0.0353	-0.4848	0.0184	0.0373	0.0960	0.1671	0.3724	1.0000	0.0147	0.0300	0.0211	-0.0028
Prm14	-0.6538	0.0311	0.0064	0.0084	0.0042	0.0073	-0.0012	0.0072	0.0147	1.0000	0.7522	0.5387	-0.0079
Prm15	-0.6299	0.0552	-0.0088	-0.0106	0.0033	-0.0017	0.0031	0.0013	0.0300	0.7522	1.0000	0.5174	-0.0160
Prm16	-0.4624	0.0617	-0.0205	0.0034	0.0176	0.0056	0.0168	0.0045	0.0211	0.5387	0.5174	1.0000	0.0013
Prm18	-0.0233	0.0265	-0.0295	0.0037	0.0105	0.0037	0.0072	-0.0067	-0.0028	-0.0079	-0.0160	0.0013	1.0000

Robbery

Parameter Information

Parameter	Effect	gender	child_gosr	seifa5	ra3	birth_cohort	rob_agg
Prm1	Intercept						
Prm2	gender	1					
Prm3	gender	0					
Prm4	child_gosr		1				
Prm5	child_gosr		0				

Parameter Information

Parameter	Effect	gender	child_gos	rseifa5	ra3	birth_cohort	rob_agg
Prm6	seifa5			2			
Prm7	seifa5			3			
Prm8	seifa5			4			
Prm9	seifa5			5			
Prm10	seifa5			1			
Prm11	ra3				2		
Prm12	ra3				3		
Prm13	ra3				1		
Prm14	birth_cohort					2	
Prm15	birth_cohort					3	
Prm16	birth_cohort					4	
Prm17	birth_cohort					1	
Prm18	rob_agg						1
Prm19	rob_agg						0

Estimated Correlation Matrix

	Prm1	Prm2	Prm4	Prm6	Prm7	Prm8	Prm9	Prm11	Prm12	Prm14	Prm15	Prm16	Prm18
Prm1	1.0000	-0.4249	-0.2588	-0.3416	-0.3084	-0.3293	-0.3537	-0.2824	-0.2034	-0.6545	-0.6312	-0.4626	-0.0049
Prm2	-0.4249	1.0000	0.1026	0.0103	-0.0115	-0.0172	-0.0063	0.0172	0.0322	0.0329	0.0585	0.0637	-0.0251
Prm4	-0.2588	0.1026	1.0000	0.1135	0.1617	0.1862	0.2007	-0.0727	-0.4877	0.0052	-0.0100	-0.0200	-0.0034
Prm6	-0.3416	0.0103	0.1135	1.0000	0.3631	0.3517	0.3359	0.1137	0.0176	0.0081	-0.0104	0.0031	-0.0075
Prm7	-0.3084	-0.0115	0.1617	0.3631	1.0000	0.3131	0.3057	0.0819	0.0375	0.0045	0.0051	0.0173	-0.0039
Prm8	-0.3293	-0.0172	0.1862	0.3517	0.3131	1.0000	0.3260	0.1623	0.0964	0.0077	-0.0006	0.0056	-0.0016
Prm9	-0.3537	-0.0063	0.2007	0.3359	0.3057	0.3260	1.0000	0.2462	0.1654	-0.0017	0.0046	0.0164	-0.0172
Prm11	-0.2824	0.0172	-0.0727	0.1137	0.0819	0.1623	0.2462	1.0000	0.3728	0.0066	0.0009	0.0041	0.0210
Prm12	-0.2034	0.0322	-0.4877	0.0176	0.0375	0.0964	0.1654	0.3728	1.0000	0.0129	0.0263	0.0176	0.0301
Prm14	-0.6545	0.0329	0.0052	0.0081	0.0045	0.0077	-0.0017	0.0066	0.0129	1.0000	0.7522	0.5389	-0.0124
Prm15	-0.6312	0.0585	-0.0100	-0.0104	0.0051	-0.0006	0.0046	0.0009	0.0263	0.7522	1.0000	0.5179	-0.0255
Prm16	-0.4626	0.0637	-0.0200	0.0031	0.0173	0.0056	0.0164	0.0041	0.0176	0.5389	0.5179	1.0000	-0.0193
Prm18	-0.0049	-0.0251	-0.0034	-0.0075	-0.0039	-0.0016	-0.0172	0.0210	0.0301	-0.0124	-0.0255	-0.0193	1.0000

Assault serious

Parameter Information

Parameter	Effect	gender	child_gosr	seifa5	ra3	birth_cohort	assault_serious
Prm1	Intercept						
Prm2	gender	1					
Prm3	gender	0					
Prm4	child_gosr		1				
Prm5	child_gosr		0				
Prm6	seifa5			2			
Prm7	seifa5			3			
Prm8	seifa5			4			
Prm9	seifa5			5			
Prm10	seifa5			1			
Prm11	ra3				2		
Prm12	ra3				3		
Prm13	ra3				1		
Prm14	birth_cohort					2	
Prm15	birth_cohort					3	
Prm16	birth_cohort					4	
Prm17	birth_cohort					1	
Prm18	assault_serious						1
Prm19	assault_serious						0

Estimated Correlation Matrix

	Prm1	Prm2	Prm4	Prm6	Prm7	Prm8	Prm9	Prm11	Prm12	Prm14	Prm15	Prm16	Prm18
Prm1	1.0000	-0.4232	-0.2579	-0.3404	-0.3074	-0.3283	-0.3533	-0.2841	-0.2063	-0.6558	-0.6333	-0.4649	-0.0791
Prm2	-0.4232	1.0000	0.1031	0.0101	-0.0117	-0.0180	-0.0074	0.0181	0.0333	0.0321	0.0569	0.0623	-0.0072
Prm4	-0.2579	0.1031	1.0000	0.1141	0.1622	0.1869	0.2004	-0.0728	-0.4874	0.0052	-0.0111	-0.0205	-0.0074
Prm6	-0.3404	0.0101	0.1141	1.0000	0.3632	0.3518	0.3360	0.1137	0.0177	0.0075	-0.0109	0.0032	0.0007
Prm7	-0.3074	-0.0117	0.1622	0.3632	1.0000	0.3133	0.3058	0.0817	0.0375	0.0042	0.0049	0.0173	0.0022
Prm8	-0.3283	-0.0180	0.1869	0.3518	0.3133	1.0000	0.3263	0.1624	0.0963	0.0074	-0.0006	0.0058	0.0046
Prm9	-0.3533	-0.0074	0.2004	0.3360	0.3058	0.3263	1.0000	0.2470	0.1665	-0.0014	0.0044	0.0173	0.0098
Prm11	-0.2841	0.0181	-0.0728	0.1137	0.0817	0.1624	0.2470	1.0000	0.3732	0.0088	0.0040	0.0064	0.0261
Prm12	-0.2063	0.0333	-0.4874	0.0177	0.0375	0.0963	0.1665	0.3732	1.0000	0.0158	0.0307	0.0211	0.0370
Prm14	-0.6558	0.0321	0.0052	0.0075	0.0042	0.0074	-0.0014	0.0088	0.0158	1.0000	0.7530	0.5401	0.0549
Prm15	-0.6333	0.0569	-0.0111	-0.0109	0.0049	-0.0006	0.0044	0.0040	0.0307	0.7530	1.0000	0.5192	0.0662
Prm16	-0.4649	0.0623	-0.0205	0.0032	0.0173	0.0058	0.0173	0.0064	0.0211	0.5401	0.5192	1.0000	0.0512
Prm18	-0.0791	-0.0072	-0.0074	0.0007	0.0022	0.0046	0.0098	0.0261	0.0370	0.0549	0.0662	0.0512	1.0000

Going armed in public to cause fear

Parameter Information

Parameter	Effect	gender	child_gosr	seifa5	ra3	birth_cohort	going
Prm1	Intercept						
Prm2	gender	1					
Prm3	gender	0					
Prm4	child_gosr		1				
Prm5	child_gosr		0				
Prm6	seifa5			2			
Prm7	seifa5			3			
Prm8	seifa5			4			
Prm9	seifa5			5			
Prm10	seifa5			1			
Prm11	ra3				2		
Prm12	ra3				3		
Prm13	ra3				1		
Prm14	birth_cohort					2	
Prm15	birth_cohort					3	
Prm16	birth_cohort					4	
Prm17	birth_cohort					1	
Prm18	going						1
Prm19	going						0

Estimated Correlation Matrix

	Prm1	Prm2	Prm4	Prm6	Prm7	Prm8	Prm9	Prm11	Prm12	Prm14	Prm15	Prm16	Prm18
Prm1	1.0000	-0.4250	-0.2591	-0.3420	-0.3083	-0.3292	-0.3536	-0.2834	-0.2039	-0.6545	-0.6312	-0.4634	-0.0027
Prm2	-0.4250	1.0000	0.1029	0.0101	-0.0120	-0.0181	-0.0077	0.0186	0.0329	0.0330	0.0583	0.0639	-0.0241
Prm4	-0.2591	0.1029	1.0000	0.1137	0.1622	0.1865	0.2001	-0.0723	-0.4875	0.0056	-0.0104	-0.0206	-0.0025
Prm6	-0.3420	0.0101	0.1137	1.0000	0.3632	0.3518	0.3361	0.1139	0.0182	0.0084	-0.0105	0.0046	-0.0013
Prm7	-0.3083	-0.0120	0.1622	0.3632	1.0000	0.3134	0.3058	0.0819	0.0375	0.0041	0.0055	0.0177	-0.0010
Prm8	-0.3292	-0.0181	0.1865	0.3518	0.3134	1.0000	0.3263	0.1628	0.0968	0.0074	-0.0007	0.0058	0.0026
Prm9	-0.3536	-0.0077	0.2001	0.3361	0.3058	0.3263	1.0000	0.2471	0.1666	-0.0016	0.0031	0.0177	0.0040
Prm11	-0.2834	0.0186	-0.0723	0.1139	0.0819	0.1628	0.2471	1.0000	0.3726	0.0074	0.0021	0.0053	0.0057
Prm12	-0.2039	0.0329	-0.4875	0.0182	0.0375	0.0968	0.1666	0.3726	1.0000	0.0132	0.0273	0.0194	0.0163
Prm14	-0.6545	0.0330	0.0056	0.0084	0.0041	0.0074	-0.0016	0.0074	0.0132	1.0000	0.7522	0.5389	-0.0112

Estimated Correlation Matrix

	Prm1	Prm2	Prm4	Prm6	Prm7	Prm8	Prm9	Prm11	Prm12	Prm14	Prm15	Prm16	Prm18
Prm15	-0.6312	0.0583	-0.0104	-0.0105	0.0055	-0.0007	0.0031	0.0021	0.0273	0.7522	1.0000	0.5178	-0.0196
Prm16	-0.4634	0.0639	-0.0206	0.0046	0.0177	0.0058	0.0177	0.0053	0.0194	0.5389	0.5178	1.0000	-0.0196
Prm18	-0.0027	-0.0241	-0.0025	-0.0013	-0.0010	0.0026	0.0040	0.0057	0.0163	-0.0112	-0.0196	-0.0196	1.0000

Act or omission with intent to harm and causing bodily harm

Parameter Information

Parameter	Effect	gender	child_gosr	seifa5	ra3	birth_cohort	act_cause
Prm1	Intercept						
Prm2	gender	1					
Prm3	gender	0					
Prm4	child_gosr		1				
Prm5	child_gosr		0				
Prm6	seifa5			2			
Prm7	seifa5			3			
Prm8	seifa5			4			
Prm9	seifa5			5			
Prm10	seifa5			1			
Prm11	ra3				2		
Prm12	ra3				3		
Prm13	ra3				1		
Prm14	birth_cohort					2	
Prm15	birth_cohort					3	
Prm16	birth_cohort					4	
Prm17	birth_cohort					1	
Prm18	act_cause						1
Prm19	act_cause						0

Estimated Correlation Matrix

	Prm1	Prm2	Prm4	Prm6	Prm7	Prm8	Prm9	Prm11	Prm12	Prm14	Prm15	Prm16	Prm18
Prm1	1.0000	-0.4250	-0.2595	-0.3422	-0.3088	-0.3296	-0.3547	-0.2834	-0.2050	-0.6538	-0.6306	-0.4618	-0.0147
Prm2	-0.4250	1.0000	0.1036	0.0105	-0.0111	-0.0171	-0.0065	0.0177	0.0334	0.0324	0.0579	0.0626	-0.0186
Prm4	-0.2595	0.1036	1.0000	0.1144	0.1625	0.1874	0.2007	-0.0733	-0.4870	0.0053	-0.0100	-0.0204	-0.0042

Estimated Correlation Matrix

	Prm1	Prm2	Prm4	Prm6	Prm7	Prm8	Prm9	Prm11	Prm12	Prm14	Prm15	Prm16	Prm18
Prm6	-0.3422	0.0105	0.1144	1.0000	0.3634	0.3520	0.3364	0.1143	0.0186	0.0075	-0.0106	0.0021	0.0156
Prm7	-0.3088	-0.0111	0.1625	0.3634	1.0000	0.3136	0.3062	0.0826	0.0381	0.0039	0.0046	0.0174	0.0094
Prm8	-0.3296	-0.0171	0.1874	0.3520	0.3136	1.0000	0.3264	0.1625	0.0961	0.0072	-0.0009	0.0053	0.0091
Prm9	-0.3547	-0.0065	0.2007	0.3364	0.3062	0.3264	1.0000	0.2474	0.1671	-0.0021	0.0037	0.0166	0.0273
Prm11	-0.2834	0.0177	-0.0733	0.1143	0.0826	0.1625	0.2474	1.0000	0.3737	0.0069	0.0021	0.0040	0.0316
Prm12	-0.2050	0.0334	-0.4870	0.0186	0.0381	0.0961	0.1671	0.3737	1.0000	0.0134	0.0271	0.0190	0.0394
Prm14	-0.6538	0.0324	0.0053	0.0075	0.0039	0.0072	-0.0021	0.0069	0.0134	1.0000	0.7521	0.5388	-0.0125
Prm15	-0.6306	0.0579	-0.0100	-0.0106	0.0046	-0.0009	0.0037	0.0021	0.0271	0.7521	1.0000	0.5182	-0.0374
Prm16	-0.4618	0.0626	-0.0204	0.0021	0.0174	0.0053	0.0166	0.0040	0.0190	0.5388	0.5182	1.0000	-0.0337
Prm18	-0.0147	-0.0186	-0.0042	0.0156	0.0094	0.0091	0.0273	0.0316	0.0394	-0.0125	-0.0374	-0.0337	1.0000
